# Anisotropy-Based Robust Performance Criteria for Linear Continuous Time Invariant Systems with Statistically Uncertain Noise

**DOI:** 10.3390/e28101157

**Published:** 2026-10-20

**Authors:** Igor G. Vladimirov

**Affiliations:** School of Engineering, Australian National University, Canberra, ACT 2601, Australia; igor.g.vladimirov@gmail.com

**Keywords:** linear stochastic system, statistically uncertain noise, root mean square gain, Tustin transform, mean anisotropy, anisotropic norm, Kullback-Leibler and Renyi relative entropy, 93C05, 93C35, 49N10, 93E20, 93B52, 93D25, 93B35, 60H10, 93E15, 47A30, 30H10, 47B35, 94A17, 60G10, 60G15, 60G44

## Abstract

This paper is concerned with robust performance analysis for continuous time invariant systems governed by linear stochastic differential equations driven by statistically uncertain Ito processes. The uncertainty is understood as the deviation of imprecisely known probability distributions of the input disturbance from those of isotropic white-noise disturbances which, up to scaling, are organized as a standard Wiener process. Using Tustin’s transform with a one-parameter family of conformal maps of the unit disk in the complex plane onto the right half-plane for discrete and continuous time transfer functions, the deviation from the nominal isotropic white-noise model is quantified by the mean anisotropy for the input of a discrete-time counterpart of the original system. The parameter of this conformal correspondence specifies the time scale for filtered versions of the input and output of the system, in terms of which the worst-case root mean square gain is formulated subject to an upper constraint on the mean anisotropy. The resulting two-parameter counterpart of the anisotropy-constrained norm of the system for the continuous time case is computed in state space by using the methods of the discrete-time anisotropy-based theory of stochastic robust filtering and control, originated by the author in the mid-1990s. These results are illustrated by an application to an electric circuit with inductive coupling and statistically uncertain noise.

## 1. Introduction

The idea of quantifying the “amplification” (or “attenuation”) properties of a linear operator from one normed space to another in terms of its induced norm is ubiquitous in functional analysis, matrix theory and numerical methods, to mention some of the relevant areas. Using such a norm for operators on Hilbert spaces underlies the $H_{\infty }$-control theory [1] for linear systems with square integrable inputs. When it is preferable to have low sensitivity of the output variables of the system to the input disturbances, the corresponding performance criteria are concerned with stabilizing the closed-loop system (thus making it a bounded input-output operator) and minimizing its operator norm by an appropriate choice of a controller [2]. This includes not only control settings as such, but also filtering problems, where the role of the output process is played by the state estimation error [3]. Due to submultiplicativity of the operator norm, its minimization leads not only to disturbance attenuation with respect to the input but also with respect to perturbations in the system itself. The small-gain theorem [4] models such perturbations as feedback loops with norm-bounded uncertainties and is closely related to the invertibility of a perturbed identity operator and von Neumann series in Banach algebras [5,6].

The Rayleigh quotient [7], whose maximization defines the induced norm of a bounded operator on a Hilbert space (or a matrix in a finite-dimensional case), reduces to a quadratic form on the unit sphere. In a generic case, its maximum over the sphere is achieved at two points, which specify the worst-case direction for the input. This direction is exceptional and is not necessarily taken by the unit vector of the normalized input disturbance. Therefore, the operator norm, as a measure of the gain, is conservative in the case when the disturbance is not targeted at specific directions. At its extreme, this suggests the averaging of the Rayleigh quotient over the uniform probability distribution on the unit sphere, which leads to an appropriately rescaled Frobenius norm of the operator. This approach is applicable only to a finite-dimensional Hilbert space, where the unit sphere has a (unique up to a multiplicative constant) finite measure which is invariant under the group of rotations (see, for example, [8,9,10] and references therein). The normalization of this hypersurface Lebesgue measure yields the uniform distribution on the sphere (while there is no uniform distribution on an infinite-dimensional sphere, which is closely related to its noncompactness).

The deviation of an arbitrary probability measure on the sphere from the uniform distribution (as a reference measure) can be quantified in terms of entropy-theoretic proximity criteria. For example, the Kullback-Leibler (KL) relative entropy [11] (with respect to the uniform distribution on the sphere) leads to the anisotropy functional [12]. If the input disturbance is random and its direction distribution is not “too far” from being uniform in the sense that its anisotropy does not exceed a given nonnegative level *a*, then the maximization of the averaged Rayleigh quotient over such distributions leads to the *a*-anisotropic norm of the matrix. This norm occupies an intermediate position between the rescaled Frobenius norm, mentioned above, and the operator norm. Moreover, the latter two norms are the extreme cases of the *a*-anisotropic norm at a=0 and as a→+∞, respectively. The advantage of this norm is that the degree of its conservativeness is controlled by the parameter *a* which specifies the amount of statistical uncertainty in the direction distribution (with the uniform distribution playing the role of a “centre” of the uncertainty class). An equivalent interpretation is that *a* quantifies how far a hypothetical opponent can go in approximating the worst-case direction (corresponding to the largest singular value of the matrix) by an absolutely continuous probability distribution on the unit sphere. Although the anisotropy functional does not lend itself to closed-form calculation even for the direction distribution of a Gaussian random vector (except for isotropic Gaussian distributions with zero mean and scalar covariance matrices, in which case the anisotropy vanishes), its asymptotic growth rate (per time step) is computable for unboundedly growing fragments of stationary Gaussian random sequences, leading to the mean anisotropy [12].

Later, the “spherical” anisotropy functional was complemented with its more tractable counterpart [13] (on the space of inputs themselves rather than the unit sphere of their directions) in the form of the minimum relative entropy of the actual probability distribution with respect to isotropic Gaussian distributions, which coincides with a multivariable version of a power-entropy construct considered in a different context and for different purposes in [14]. Although this second anisotropy functional is an upper bound for the original one, it has the same infinite-horizon growth rate in the stationary Gaussian case mentioned above, with the resulting mean anisotropy being expressed in terms of the spectral density and the mean value of the sequence. Accordingly, the averaged Rayleigh quotient was replaced with the ratio of root-mean-square (RMS) values of the output and input.

The anisotropy functionals, anisotropy-constrained norms and anisotropy-based theory of stochastic robust control and filtering for linear systems (without internal perturbations) in infinite-horizon time-invariant and finite-horizon time-varying settings, as well as for Toeplitz operators acting on homogenous Gaussian random fields on multidimensional lattices, were originated by the author in a series of papers and research reports in the mid 1990s–early 2000s, starting from [12] (and its subsequently written conference version [15]) and including [16,17,18,19,20] whose extended account was provided in [21] and followed by [13,22,23,24]. Those founding works also included a set of functions (written in MATLAB 5.0) for anisotropy-based robust performance analysis and controller design reported in [16]. Being motivated as a stochastic extension of the $H_{\infty }$-control theory to statistically uncertain linear discrete-time systems, the above mentioned developments aimed to bridge the gap between the deterministic approach of $H_{\infty }$-control and the stochastic paradigm of linear quadratic Gaussian (LQG) control, which includes Kalman filtering [25] and its predecessor—the Kolmogorov-Wiener-Hopf theory of smoothing, filtering and prediction for stationary random processes [26,27]. This extension also addressed the robustness issues of the LQG approach which is oriented to an idealized scenario, when the random input disturbances have precisely known statistical characteristics and are organized as a Gaussian white-noise sequence or a standard Wiener process (from which a more complicated covariance structure can be obtained by using a shaping filter).

Not discussing here the other ways of combining the $H_{\infty }$-theory and LQG approaches (see, for example, [28]), we note that the anisotropy-based theory offers probabilistic, system theoretic and computational tools (including a homotopy method for solving specific sets of cross-coupled Riccati, Lyapunov and log-determinant matrix algebraic equations) for robust performance analysis and synthesis of controllers and filters. This approach addresses robustness with respect to spatially and temporally coloured random disturbances (in the sense of statistical correlations between the entries of a multivariable noise at the same or different moments of time) with imprecisely known statistical properties. This is achieved by quantifying the statistical uncertainty (as a deviation from the nominal Gaussian white noise model) in terms of anisotropy, so that the resulting anisotropy-constrained version of the induced operator norm describes the worst-case RMS gain of the system over this uncertainty class.

Subsequently (in the 2000s and more recently), the theory was also being developed in the form of a suboptimal guaranteed approach to systems with internal perturbations [29], towards convexification of the anisotropy-based control synthesis [30,31,32] and suboptimal observer design [33,34], control settings with nonzero-mean input disturbances [35] and multiplicative noise [36,37,38] in the system, estimation problems with sensor failures [39], roundoff noise effects in digital controllers [40], parameterization of anisotropy-based controllers [41] and their asymptotic behaviour at low mean anisotropy levels [42], as well as other developments, including an adaptation of the anisotropy-based theory to descriptor systems [43].

The discrete-time setting is essential for the anisotropy-based theory, which stems from the anisotropy functionals using finite-dimensional spheres and finite segments of random sequences. At the same time, entropy-theoretic constructs are also known for infinite-dimensional objects such as diffusion processes. For example, the relative entropy (with respect to the Wiener measure) is correctly defined for the probability distribution of a diffusion process governed by a stochastic differential equation (SDE) with a nonzero drift (satisfying Novikov’s condition [44]) and the identity diffusion matrix. This is due to Girsanov’s theorem [45], where the preservation of the diffusion matrix of the reference standard Wiener process (so that only the drift is changed) is essential for the absolute continuity of measures, which plays an important role in relative entropy formulations of statistical uncertainty for continuous time stochastic control systems driven by diffusion processes [46,47,48].

The present paper (whose brief preprint version was archived in [49]) provides one of possible ways of rigorously extending the anisotropy-based approach (which underlies stochastic minimax formulations of robust filtering and control using worst-case RMS gains under entropy-theoretic constraints in terms of anisotropy) to linear continuous time invariant (LCTI) systems driven by statistically uncertain Ito processes. To this end, we employ a parametric family of conformal maps (specified by an auxiliary time scale parameter and related to the Cayley transform [50]) between the unit disk and the right half-plane for the discrete and continuous time transfer functions, respectively. Up to a multiplicative constant, these maps are identical to Tustin’s transform [51] for converting LCTI systems to linear discrete-time invariant (LDTI) systems and the other way around. This results in a subsidiary LDTI system which lends itself to application of the anisotropy-based theory. The spectral densities of the input and output of this subsidiary system are related to those of filtered versions of the original continuous-time processes (with the time scale parameter specifying the transient time of the filter) due to the relation between the spectral density of the Ornstein-Uhlenbeck (OU) process [52] and the logarithmic derivative of the Cayley map. The conformal correspondence is closely related to the presence of a denominator (which secures integrability in the continuous time case) in the inner-outer factorizability condition for spectral densities (describing the physical realizability of such a density with the aid of a stable causal shaping filter) [53,54]. This is one of the reasons why discrete-time results (many of which rely on boundedness of the frequency range) cannot merely be adopted without due modification (in particular, without taking the above mentioned denominator into account) for the continuous-time case, where the processes may contain arbitrarily fast components accommodated by the infinite frequency range making certain integrals divergent. The conformal relation allows the RMS gain of the LCTI system output with respect to the input noise to be defined in terms of the filtered processes. The resulting continuous-time extension of the anisotropic norm is the largest value of the gain subject to an upper constraint on the mean anisotropy of the noise as an entropy-theoretic measure of its deviation from isotropic white-noise disturbances which, up to scaling, are organized as the standard Wiener process. We also discuss the probabilistic and information-theoretic aspects of the worst-case disturbance which inherits from its discrete-time counterpart the structure of an additive mixture of a state-dependent drift with an innovation martingale process.

The paper is organized as follows. Section 2 describes the class of LCTI systems under consideration governed by linear SDEs driven by random disturbances in the form of Ito processes. Section 3 specifies the class of random disturbances produced by LCTI noise shaping filters from a standard Wiener process. Section 4 describes filtered versions of the underlying processes for such a system. Section 5 defines the RMS gain of the system in terms of the filtered input and output. Section 6 calculates the RMS gain in the nominal case of isotropic white-noise disturbances. Section 7 specifies the parameter-dependent conformal correspondence to a discrete-time system with the same RMS gain. Section 8 defines a two-parameter norm of the underlying system in terms of the anisotropic norm of the effective LDTI system. Section 9 describes state-space equations for computing this continuous-time extension of the anisotropic norm along with the structure of the worst-case disturbance. Section 10 discusses the Renyi [55] and KL relative entropy rates for the worst-case noise with respect to its martingale part and links them with the temporal component of the mean anisotropy level. Section 11 provides a numerical example of computing the two-parameter continuous-time extension of the anisotropic norm for an RLC circuit with coupled inductors and statistically uncertain noise. Section 12 makes concluding remarks.

## 2. Linear Stochastic Systems Being Considered

Consider an LCTI system *F* with an input *W*, which represents the external noise acting on the system, and an output *Z*. The latter consists of variables whose small values are beneficial for the system performance. For example, *Z* can be formed from reference trajectory tracking errors or state estimation errors which have to be minimized in some sense. It is assumed that *W* and *Z* are Ito processes [52] (with respect to a common filtration on the underlying probability space) on the real time axis, which take values in $R^{m}$ and $R^{p}$, respectively, and are related in a causal fashion as

(1) $$Z\left(t\right)=\int_{-\infty }^{t}f\left(t-\tau \right)dW\left(\tau \right)+f_{0}W\left(t\right),\;t\in R.$$

Here, $f_{0}\in R^{p\times m}$ is a static gain matrix, and $f:R_{+}\to R^{p\times m}$ is the step response of the system as a linear input-output operator W↦Z, with $R_{+}:=\left[0,+\infty \right)$. In particular, if the noise *W* is a standard Wiener process [52] in $R^{m}$, then the Ito integral in (1) is well-defined for any square integrable function $f\in L^{2}\left(R_{+},R^{p\times m}\right)$. In this case, the Ito isometry yields

(2) $$E\left(|\int_{-\infty }^{t}f\left(t-\tau \right)dW\left(\tau \right){|}^{2}\right)=\int_{R_{+}}{∥f\left(\tau \right)∥}_{F}^{2}d\tau <+\infty$$

for any time t∈R, where E(·) is expectation, and ${∥\cdot ∥}_{F}$ is the Frobenius norm of matrices [7]. The increments of such a process *W* have the identity covariance operator on the Hilbert space $L^{2}\left(R,R^{m}\right)$ in the sense that the bilinear form

(3) $$H\left(\varphi ,\psi \right):=E\left(\int_{R}\varphi {\left(s\right)}^{T}dW\left(s\right)\int_{R}\psi {\left(t\right)}^{T}dW\left(t\right)\right)$$

reduces to the usual inner product

(4) $$H\left(\varphi ,\psi \right)=\int_{R}\varphi {\left(t\right)}^{T}\psi \left(t\right)dt,\;\varphi ,\psi \in L^{2}\left(R,R^{m}\right),$$

with (2) being a corollary of (4). Otherwise, that is, when *W* is not a standard Wiener process, its covariance structure can be more complicated, and the equalities (2) and (4) are no longer valid.

If f(τ) is a linear combination of quasi-polynomials [56] which decay exponentially fast, as τ→+∞, then there exist constant matrices

(5) $$A\in R^{n\times n},\;B\in R^{n\times m},\;C\in R^{p\times n},$$

with *A* Hurwitz, such that

(6) $$f\left(\tau \right)=CA^{-1}e^{\tau A}B,\;\tau ⩾0.$$

Then the Ito stochastic differential of the system output process *Z* in (1) acquires the form

(7) $$\begin{array}{cc} dZ(t)\; & =\left(\int_{-\infty }^{t}f^{\prime }\left(t-\tau \right)dW\left(\tau \right)\right)dt+\left(f\left(0\right)+f_{0}\right)dW\left(t\right)\\ \; & =CX(t)dt+DdW(t), \end{array}$$

where

(8) $$X\left(t\right):=\int_{-\infty }^{t}e^{(t-\tau )A}BdW\left(\tau \right),$$

and

(9) $$D:=f_{0}+CA^{-1}B$$

is the feedthrough (p×m)-matrix. The relation (7), which can also be obtained by using the integration by parts

$$\int_{-\infty }^{t}f\left(t-\tau \right)dW\left(\tau \right)=f\left(0\right)W\left(t\right)+\int_{-\infty }^{t}f^{\prime }\left(t-\tau \right)W\left(\tau \right)d\tau ,$$

shows that the corresponding impulse response of the system is given by the time derivative of (6):

(10) $$f^{\prime }\left(\tau \right)=Ce^{\tau A}B,\;\tau ⩾0.$$

The $R^{n}$-valued Ito process *X* in (8) is an internal state of the system *F* and satisfies the linear SDE

(11) dX(t)=AX(t)dt+BdW(t)

whose solution on a time interval [s,+∞), with an arbitrary start time s∈R, is given by

(12) $$X\left(t\right)=e^{(t-s)A}X\left(s\right)+\int_{s}^{t}e^{(t-\tau )A}BdW\left(\tau \right),\;t⩾s.$$

As opposed to the finite-dimensional setting (5)–(12), the system state can, in general, be essentially infinite-dimensional, as in the case of systems with delay [57]. For example, such a system arises if the drift AX(t) in (11) is replaced with a linear function of the past history of the process *X*:

(13) $$dX\left(t\right)=\left(\int_{R_{+}}\vartheta \left(d\tau \right)X\left(t-\tau \right)\right)dt+BdW\left(t\right),$$

where ϑ is an $R^{n\times n}$-valued countably additive measure on the σ-algebra of Borel subsets of $R_{+}$, and the integral is understood in the pathwise sense. The SDE (11) is a particular case of (13) with an atomic measure ϑ concentrated at the origin as $\vartheta \left(S\right)=\chi_{S}\left(0\right)A$, where $\chi_{S}$ is the indicator function of a set *S*. More generally, if ϑ is of bounded support, so that suppϑ⊂[0,r] for some r⩾0, then the effective state of the system at time *t* is the past history of *X* over the time interval [t−r,t].

Returning to the SDEs (7) and (11), we note that there also are alternative ways to model the input-output operator. For example, instead of (7), the output channel can be (and often is) described by a different relation:

Z=CX.

However, in view of (11), it can also be represented in the form (7) with appropriately modified matrices as

$$dZ=\tilde{C}Xdt+DdW,\;\tilde{C}:=CA,\;D:=CB.$$

Furthermore, a convenient feature of the representation (7) is that both the input *W* and the output *Z* enter the SDEs (7) and (11) in a unified fashion (through their Ito increments), thus allowing such systems to be easily concatenated.

## 3. Random Disturbances Under Consideration

In what follows, we will be concerned with a setting where the $R^{m}$-valued input *W* of the system *F* does not necessarily obey the nominal model assumption of being a standard Wiener process. Rather, *W* can be a more general Ito process with respect to a standard Wiener process *V* in $R^{m}$ as the primary source of randomness. Therefore, the $R^{n}$-valued state *X* and the $R^{p}$-valued output *Z*, which are driven by *W* according to the SDEs (7) and (11), are also Ito processes with respect to *V*.

More precisely, the process *W* is assumed to be the output of a causal LCTI system *G* playing the role of a noise shaping filter with a static gain matrix $g_{0}\in R^{m\times m}$ and a square integrable step response $g\in L^{2}\left(R_{+},R^{m\times m}\right)$:

(14) $$W\left(t\right)=\int_{-\infty }^{t}g\left(t-\tau \right)dV\left(\tau \right)+g_{0}V\left(t\right),$$

see Figure 1.

Similarly to the system *F*, the filter *G* can also have a finite-dimensional state-space realization

(15) dξ=αξdt+βdV,dW=cξdt+ddV

(the time arguments are omitted for the sake of brevity), where ξ is an $R^{\nu }$-valued Ito process, and

(16) $$\alpha \in R^{\nu \times \nu },\;\beta \in R^{\nu \times m},\;c\in R^{m\times \nu },\;d\in R^{m\times m},$$

with α Hurwitz. In this case, similarly to (6) and (9), the static gain matrix and the step response in (14) are given by

(17) $$g_{0}=d-c\alpha^{-1}\beta ,\;g\left(\tau \right)=c\alpha^{-1}e^{\tau \alpha }\beta ,\;\tau ⩾0.$$

The presence of the drift cξ in (15) makes the noise process *W* temporally “coloured”, so that its increments on disjoint time intervals acquire statistical dependence. Also, nonzero off-diagonal entries of the diffusion matrix $dd^{T}$ (which, in general, is nondiagonal) make the Ito increment of *W* spatially correlated in the sense of dependence between its components. Moreover, the combined effect of the drift and diffusion can lead to dependence between the components of *W* itself.

We denote the state-space realizations of the system *F* in (7) and (11) and the noise shaping filter *G* in (15) as

$$F↔\left[\begin{array}{cc} A & B\\ C & D \end{array}\right],\;G↔\left[\begin{array}{cc} \alpha & \beta \\ c & d \end{array}\right].$$

**Figure 1:**
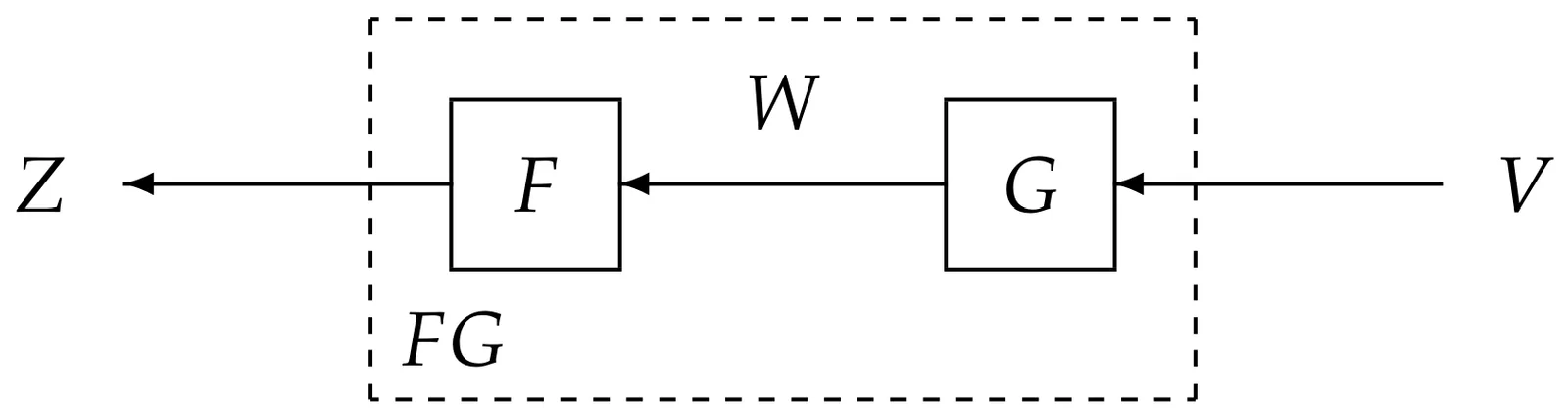
The LCTI system *F* with the output *Z* and input *W* produced by a causal LCTI noise shaping filter *G* from the standard Wiener process *V*.

The resulting LCTI system FG in Figure 1 has the same output *Z* as the system *F* and is driven by the standard Wiener process *V* at the input of the noise shaping filter *G*. Its equivalent state-space realizations

(18) $$FG↔\left[\begin{array}{ccc} \alpha & 0 & \beta \\ Bc & A & Bd\\ Dc & C & Dd \end{array}\right]↔\left[\begin{array}{ccc} A & Bc & Bd\\ 0 & \alpha & \beta \\ C & Dc & Dd \end{array}\right]$$

are expressed in terms of the matrices (5), (9) and (16) and involve the corresponding augmented state processes

$$\left[\begin{array}{c} \xi \\ X \end{array}\right],\;\left[\begin{array}{c} X\\ \xi \end{array}\right].$$

If not only the system *F* is known precisely, but so also is the noise shaping filter *G*, then the coloured noise setting can be reduced to the case of the standard Wiener process *V* at the input by replacing *F* with FG and thus incorporating the dynamics of the system *F* and that of the external disturbance *W* in the augmented state space $R^{n+\nu }$. Therefore, the colouredness of *W* as such does not lead to statistical uncertainty. Actual uncertainty arises when *G* is known imprecisely, in which case, the augmented system FG acquires internal uncertainty.

Note that there is an important class of noise shaping filters *G*, which depend on the system *F* and do not lead to the state augmentation in FG mentioned above. More precisely, if *G* is organized as a “parasitic” feedback specified by matrices $L\in R^{m\times n}$ and $M\in R^{m\times m}$ as

**Figure 2:**
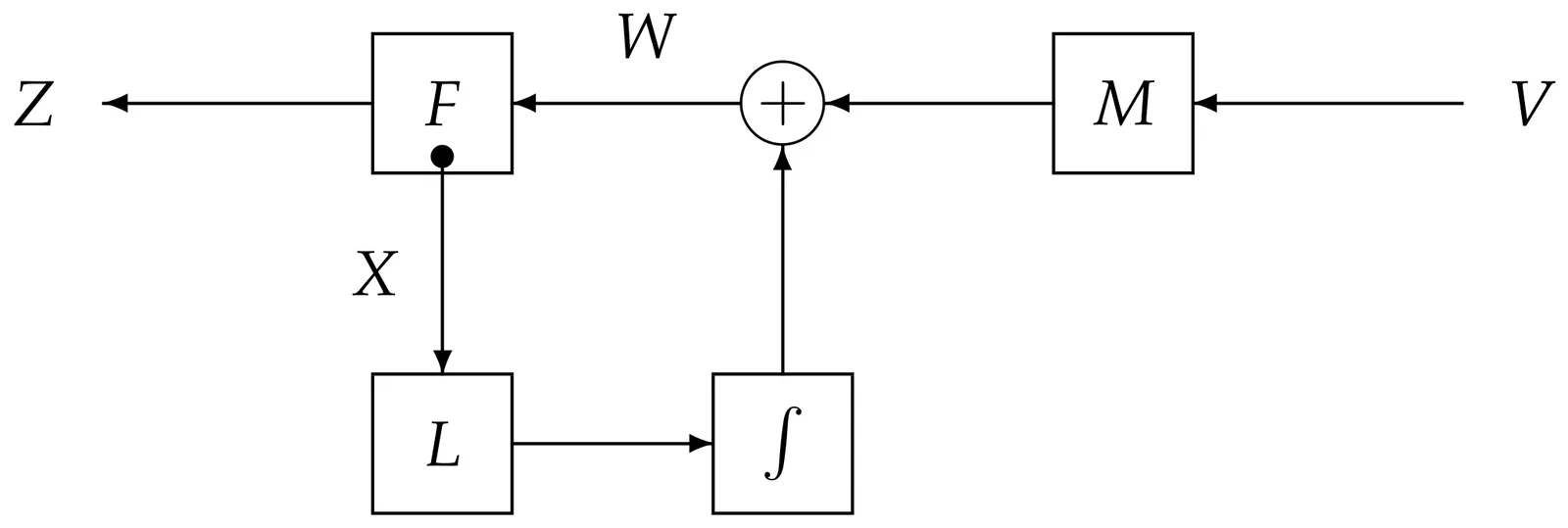
The noise shaping filter *G* described by (19) exploits the internal state *X* of the system *F* through the feedback loop specified by the matrix *L*, which leads to a drift in the resulting disturbance *W*.

(19) dW=LXdt+MdV,

whereby the internal state *X* of the system *F* gives rise to the drift LX in the disturbance *W* (see Figure 2), then substitution of (19) into (7) and (11) yields

(20) $$FG↔\left[\begin{array}{cc} A+BL & BM\\ C+DL & DM \end{array}\right].$$

In this case, the system *F* and the noise shaping filter *G* share the common state *X* in $R^{n}$, thus making the state-space realizations (18) nonminimal. Despite being special, the noise structure (19) is typical for the worst-case random disturbances in the framework of anisotropy-based approach to system robustness with respect to statistically uncertain noise. This is discussed in Section 8 and Section 9 and related to its discrete-time counterpart [18] through the conformal correspondence described in Section 7. Also, the above example blurs the line between internal uncertainties in the system and uncertainties in the dynamics of external disturbances.

Being a Gaussian Markov process with almost surely continuous sample paths and independent stationary increments with the covariance matrix

(21) $$\mathrm{cov}\left(V\left(s\right)-V\left(t\right)\right)=\left|s-t\right|I_{m},\;s,t\in R,$$

in accordance with the Ito isometry (here, $I_{m}$ is the identity matrix of order *m*), the standard Wiener process *V* has almost surely nowhere differentiable sample paths of unbounded variation and nonzero quadratic variation over any nondegenerate time interval [52]. Such nonsmoothness is inherited by the Ito processes *Z*, *X*, *W* in (7), (8) and (14). Furthermore, in the case of nonzero static gain matrices $f_{0}$, $g_{0}$, the variances of *Z*, *W* grow unboundedly over time (despite the stability of the system *F* and the noise shaping filter *G* due to their dynamics matrices *A*, α being Hurwitz). We will therefore consider filtered versions of these processes.

## 4. Filtered Processes and Transfer Functions

As a more regular (in the sense of the variances) representation of the input and output processes *V*, *W*, *Z* of the system *F* and the noise shaping filter *G* in Figure 1, consider their filtered versions $V_{T}$, $W_{T}$, $Z_{T}$ governed by SDEs

(22) $$\begin{array}{cc} dV_{T}\; & =-\Omega V_{T}dt+\sqrt{2\Omega }dV, \end{array}$$

(23) $$\begin{array}{cc} dW_{T}\; & =-\Omega W_{T}dt+\sqrt{2\Omega }dW, \end{array}$$

(24) $$\begin{array}{cc} dZ_{T}\; & =-\Omega Z_{T}dt+\sqrt{2\Omega }dZ. \end{array}$$

Here, T>0 is an auxiliary time scale parameter, and use is also made of a related frequency parameter

(25) $$\Omega :=\frac{1}{T}.$$

Note that the common filter in (22)–(24), parameterized by *T* (or Ω), does not affect the underlying system *F* or the noise shaping filter *G* which remain the same input-output operators W↦Z and V↦W, respectively. Rather, it provides an alternative way for describing the properties of *F* and *G* in terms of the filtered processes $V_{T}$, $W_{T}$, $Z_{T}$ instead of the original processes *V*, *W*, *Z*, as discussed in what follows.

With *V* being a standard Wiener process (to which *W* in (14) reduces when g=0, $g_{0}=I_{m}$), the SDE (22) has a unique invariant measure in the form of the standard normal distribution and generates an OU process [52] in $R^{m}$. The latter is a zero-mean stationary Gaussian diffusion process with the covariance function

(26) $$\mathrm{cov}\left(V_{T}\left(s\right),V_{T}\left(t\right)\right)=e^{-\Omega |s-t|}I_{m}=e^{-|s-t|/T}I_{m},\;s,t\in R,$$

so that the variance

$$E(|V_{T}\left(t\right){|}^{2})=m$$

remains constant, and *T* quantifies the correlation time of the OU process in the sense that the covariance function (26) decays by a factor of e (that is, $\mathrm{cov}\left(V_{T}\left(s\right),V_{T}\left(t\right)\right)=\frac{1}{e}I_{m}$) whenever |s−t|=T. However, since we will be concerned with the infinite-horizon asymptotic behaviour of stable systems, it is convenient to endow (22)–(24) with zero initial conditions

(27) $$V_{T}\left(0\right)=0,\;W_{T}\left(0\right)=0,\;Z_{T}\left(0\right)=0.$$

Then the filtered processes $V_{T}$, $W_{T}$, $Z_{T}$ are represented in terms of *V*, *W*, *Z* at any time t⩾0 as

(28) $$\begin{array}{cc} V_{T}\left(t\right)\; & =\int_{0}^{t}\phi \left(t-\tau \right)dV\left(\tau \right), \end{array}$$

(29) $$\begin{array}{cc} W_{T}\left(t\right)\; & =\int_{0}^{t}\phi \left(t-\tau \right)dW\left(\tau \right), \end{array}$$

(30) $$\begin{array}{cc} Z_{T}\left(t\right)\; & =\int_{0}^{t}\phi \left(t-\tau \right)dZ\left(\tau \right), \end{array}$$

with

(31) $$\phi \left(\tau \right):=\sqrt{2\Omega }e^{-\Omega \tau },\;\tau ⩾0,$$

which makes them only asymptotically stationary in the long run. Note that the right-hand sides of (28)–(30) are organized as weighted moving averages of the Ito increments of the corresponding processes *V*, *W*, *Z*. Similarly to (26), the weighting kernel function ϕ(τ) in (31) decays by a factor of ϕ(0)/ϕ(T)=e at τ=T in view of (25), so that the time scale parameter *T* plays the role of an effective averaging window length (or effective memory length) of the filter. In particular, the “long-memory” asymptotic behaviour of the filtered processes is described by

(32) $$\begin{array}{c} \sqrt{\frac{T}{2}}V_{T}\left(t\right)\to V(t)-V(0), \end{array}$$

(33) $$\begin{array}{c} \sqrt{\frac{T}{2}}W_{T}\left(t\right)\to W(t)-W(0), \end{array}$$

(34) $$\begin{array}{c} \sqrt{\frac{T}{2}}Z_{T}\left(t\right)\to Z(t)-Z(0),\;\mathrm{as}\;T\to +\infty , \end{array}$$

where the convergence holds for any given t⩾0 in the mean-square sense. The latter is verified by using the covariance operators for the Ito increments of *V*, *W*, *Z*. For example, in application to (28), it is established by

(35) $$\begin{array}{cc} E\left(|\sqrt{\frac{T}{2}}V_{T}\left(t\right)-V\left(t\right)+V\left(0\right){|}^{2}\right)\; & =E\left(|\int_{0}^{t}\left(e^{\Omega (\tau -t)}-1\right)dV\left(\tau \right){|}^{2}\right)\\ \; & =m\int_{0}^{t}{\left(1-e^{-\Omega \tau }\right)}^{2}d\tau \\ \; & ∼\frac{mt^{3}}{3T^{2}}\to 0,\;\mathrm{as}\;T\to +\infty , \end{array}$$

where the Ito isometry for the $R^{m}$-valued standard Wiener process *V* is combined with (25). The convergence in (32)–(34) also holds almost surely, which can be obtained by using the integration by parts as

(36) $$\begin{array}{cc} \sqrt{\frac{T}{2}}V_{T}\left(t\right)-V\left(t\right)+V\left(0\right)\; & =\int_{0}^{t}\left(e^{\Omega (\tau -t)}-1\right)dV\left(\tau \right)\\ \; & =\left(\left(e^{\Omega (\tau -t)}-1\right)V\left(\tau \right)\right){|}_{\tau =0}^{t}-\Omega \int_{0}^{t}e^{\Omega (\tau -t)}V\left(\tau \right)d\tau \\ \; & =\left(1-e^{-\Omega t}\right)V\left(0\right)-\Omega \int_{0}^{t}e^{\Omega (\tau -t)}V\left(\tau \right)d\tau \\ \; & \overset{a.s.}{∼}\frac{1}{T}\left(tV\left(0\right)-\int_{0}^{t}V\left(\tau \right)d\tau \right)\overset{a.s.}{⟶}0,\;\mathrm{as}\;T\to +\infty , \end{array}$$

and in a similar fashion for the processes $W_{T}$, $Z_{T}$. Both the mean-square convergence in (35) and the almost sure convergence in (36) are uniform over a bounded time interval 0⩽t⩽b for any given b>0. Concerning the “short-memory” (as T→0+) asymptotic behaviour, it is discussed below for filtered state processes and also (along with the case of T→+∞) in Section 6 for the RMS gain of the system *F* defined in Section 5 in terms of $W_{T}$, $Z_{T}$ from (29) and (30).

The asymptotic input-output properties of the system *F*, governed by (7) and (11) with a Hurwitz dynamics matrix *A*, and those of the noise shaping filter *G* in (14) can be formulated in terms of the transfer functions

(37) $$F\left(s\right):=C{\left(sI_{n}-A\right)}^{-1}B+D$$

(analytic in a neighbourhood of the closed right half-plane C+:={s∈C:Res⩾0}), and

(38) $$G\left(s\right):=\int_{R_{+}}e^{-st}g^{\prime }\left(t\right)dt+d=s\int_{R_{+}}e^{-st}g\left(t\right)dt+g_{0}$$

(with a slight abuse of notation, we identify the LCTI systems under consideration with their transfer functions). Here, for simplicity, the noise shaping filter *G* is also assumed to be a stable system with a finite-dimensional state ξ, described by (15)–(17), so that (38) is a rational function

(39) $$G\left(s\right)=c{\left(sI_{\nu }-\alpha \right)}^{-1}\beta +d,$$

analytic in a neighbourhood of $C_{+}$. The transfer function *G* factorizes the spectral density $S:R\to H_{m}^{+}$ for the Gaussian process *W* from (15) with zero-mean stationary increments as

(40) $$S\left(\omega \right):=G\left(i\omega \right)G{\left(i\omega \right)}^{*},\;\omega \in R,$$

where $H_{m}^{+}$ is the set of complex positive semi-definite Hermitian matrices of order *m*, and ${\left(\cdot \right)}^{*}:={\left(\bar{\left(\cdot \right)}\right)}^{T}$ is the complex conjugate transpose. Their covariance structure, described by (3), is represented as

(41) $$H\left(\varphi ,\psi \right)=\frac{1}{2\pi }\int_{R}\hat{\varphi }{\left(\omega \right)}^{*}S\left(\omega \right)\hat{\psi }\left(\omega \right)d\omega$$

for any functions $\varphi ,\psi \in L^{2}\left(R,R^{m}\right)$ in terms of the Fourier transforms

$$\hat{\varphi }\left(\omega \right):=\int_{R}e^{-i\omega t}\varphi \left(t\right)dt,\;\hat{\psi }\left(\omega \right):=\int_{R}e^{-i\omega t}\psi \left(t\right)dt,$$

with the nominal standard Wiener process model (4) being a particular case of (41) when $S=I_{m}$.

Similarly to deterministic linear systems, the transfer functions *F*, *G* specify the linear relations

(42) $$\hat{Z}\left(s\right)=F\left(s\right)\hat{W}\left(s\right),\;\hat{W}\left(s\right)=G\left(s\right)\hat{V}\left(s\right)$$

between the Laplace transforms of the input and output in the case of zero initial states X(0)=0 and ξ(0)=0:

(43) $$\begin{array}{cc} \hat{V}\left(s\right)\; & :=\int_{R_{+}}e^{-st}dV\left(t\right), \end{array}$$

(44) $$\begin{array}{cc} \hat{W}\left(s\right)\; & :=\int_{R_{+}}e^{-st}dW\left(t\right), \end{array}$$

(45) $$\begin{array}{cc} \hat{Z}\left(s\right)\; & :=\int_{R_{+}}e^{-st}dZ\left(t\right), \end{array}$$

where the Ito integrals are well-defined for any s∈C with Res>0. The same relations, as in (42),

(46) $${\hat{Z}}_{T}\left(s\right)=F\left(s\right){\hat{W}}_{T}\left(s\right),\;{\hat{W}}_{T}\left(s\right)=G\left(s\right){\hat{V}}_{T}\left(s\right)$$

hold for the usual Laplace transforms (as opposed to the Ito incremental ones in (43)–(45))

(47) $$\begin{array}{cc} {\hat{V}}_{T}\left(s\right)\; & :=\int_{R_{+}}e^{-st}V_{T}\left(t\right)dt=\Phi \left(s\right)\hat{V}\left(s\right), \end{array}$$

(48) $$\begin{array}{cc} {\hat{W}}_{T}\left(s\right)\; & :=\int_{R_{+}}e^{-st}W_{T}\left(t\right)dt=\Phi \left(s\right)\hat{W}\left(s\right), \end{array}$$

(49) $$\begin{array}{cc} {\hat{Z}}_{T}\left(s\right)\; & :=\int_{R_{+}}e^{-st}Z_{T}\left(t\right)dt=\Phi \left(s\right)\hat{Z}\left(s\right) \end{array}$$

of the filtered processes $V_{T}$, $W_{T}$, $Z_{T}$ in (28)–(30) as illustrated by Figure 3, where the transfer function

(50) Φ(s):=∫R+e−stϕ(t)dt=2Ωs+Ω=2T1+Ts,Res>−Ω,

specifies a low-pass filter with the impulse response ϕ in (31) and the cutoff frequency Ω in (25).

Indeed, in view of (47)–(50), the relations (46) are equivalent to (42) since the scalar-valued function Φ is nonzero everywhere and commutes with any transfer matrix. In the linear time invariant setting, this commutativity is equivalent to the commutativity of the corresponding input-output operators.

Note that, although the filtered processes $Z_{T}$, $W_{T}$, $V_{T}$ are related by the same input-output operators *F*, *G* as *Z*, *W*, *V*, their dynamics (represented by the lower part of the diagram in Figure 3) are governed in state space by pathwise ordinary differential equations (ODEs)

(51) $$\begin{array}{cc} {\dot{X}}_{T}\; & =AX_{T}+BW_{T},\;Z_{T}=CX_{T}+DW_{T}, \end{array}$$

(52) $$\begin{array}{cc} {\dot{\xi }}_{T}\; & =\alpha \xi_{T}+\beta V_{T},\;\;W_{T}=c\xi_{T}+dV_{T}, \end{array}$$

where $\dot{\left(\;\right)}$ is the time derivative. Accordingly, the filtered state processes $X_{T}$, $\xi_{T}$ have almost surely continuously differentiable sample paths and are related to the original states *X*, ξ (see the upper part of the diagram in Figure 3) by the ODEs

(53) $${\dot{X}}_{T}=-\Omega X_{T}+\sqrt{2\Omega }X,\;{\dot{\xi }}_{T}=-\Omega \xi_{T}+\sqrt{2\Omega }\xi ,$$

where the low-pass filter Φ in the usual ODE form (instead of the incremental SDE structure) does provide a smoothing effect. These ODEs are also endowed with zero initial conditions

(54) $$X_{T}\left(0\right)=0,\;\xi_{T}\left(0\right)=0.$$

In the nominal white noise regime, when *W* reproduces the standard Wiener process *V*, so that c=0, $d=I_{m}$ in (15), and $W_{T}=V_{T}$ in accordance with the second equality in (52), the filtered system dynamics (51) are driven by the OU process $V_{T}$.

The above mentioned smoothing effect manifests itself in that, upon appropriate scaling, the filtered state processes $X_{T}$, $\xi_{T}$ asymptotically reproduce the original state processes *X*, ξ in the sense that

(55) $$\frac{1}{\sqrt{2T}}\left[\begin{array}{c} X_{T}\left(t\right)\\ \xi_{T}\left(t\right) \end{array}\right]=\Omega \int_{0}^{t}e^{\Omega (\tau -t)}\left[\begin{array}{c} X(\tau )\\ \xi (\tau ) \end{array}\right]d\tau \overset{a.s.}{⟶}\left[\begin{array}{c} X(t)\\ \xi (t) \end{array}\right],\;\mathrm{as}\;T\to 0+,$$

with Ω depending on *T* according to (25). Here, the almost sure convergence to X(t), ξ(t) holds pointwise for any fixed but otherwise arbitrary t>0 since *X*, ξ have almost surely continuous sample paths (which are therefore bounded on any finite time interval) and due to the weak convergence [58] of the exponential distribution with the probability density function (PDF)

$$\sqrt{\frac{\Omega }{2}}\phi \left(\tau \right)=\Omega e^{-\Omega \tau },\;\tau ⩾0$$

(arising from (31)) to the atomic probability measure on Borel subsets of $R_{+}$ concentrated at the origin 0, as Ω→+∞. The pointwise (with respect to time *t*) convergence to the almost surely continuous processes *X* and ξ in (55) can also be established as a corollary of the relations

(56) $$\begin{array}{cc} |\Omega \int_{0}^{t}e^{\Omega (\tau -t)}\psi \left(\tau \right)d\tau -\psi \left(t\right)|= & \left|\int_{0}^{\Omega t}e^{-v}\left(\psi \left(t-Tv\right)-\psi \left(t\right)\right)dv-e^{-\Omega t}\psi \left(t\right)\right|\\ ⩽ & \left|\int_{0}^{\epsilon \Omega t}e^{-v}\left(\psi \left(t-Tv\right)-\psi \left(t\right)\right)dv\right|\\ \; & +|\int_{\epsilon \Omega t}^{\Omega t}e^{-v}\left(\psi \left(t-Tv\right)-\psi \left(t\right)\right)dv|+e^{-\Omega t}\left|\psi \left(t\right)\right|\\ ⩽ & \max_{\delta \in [0,\epsilon ]}\left|\psi \left(\left(1-\delta \right)t\right)-\psi \left(t\right)\right|\\ \; & +2\left(e^{-\epsilon \Omega t}-e^{-\Omega t}\right)\max_{\tau \in [0,t]}|\psi \left(\tau \right)|+e^{-\Omega t}\left|\psi \left(t\right)\right|\\ ⩽ & \max_{\delta \in [0,\epsilon ]}|\psi \left(\left(1-\delta \right)t\right)-\psi \left(t\right)|+2e^{-\epsilon \Omega t}\max_{\tau \in [0,t]}\left|\psi \left(\tau \right)\right|, \end{array}$$

which hold for any T>0, ϵ∈[0,1], t⩾0 and any continuous function $\psi \in C(R_{+},R^{r})$. Indeed, by using, for example,

(57) $$\epsilon :=\frac{2\sqrt{\Omega t}}{1+\Omega t}$$

which, in view of (25), satisfies

(58) ϵ→0,ϵΩt→+∞,asT→0+,

for any given t>0, it follows that the corresponding right-hand side of (56) has zero limit by the continuity of ψ, and hence,

(59) $$\lim_{T\to 0+}\left(\Omega \int_{0}^{t}e^{\Omega (\tau -t)}\psi \left(\tau \right)d\tau \right)=\psi \left(t\right),$$

thereby establishing the pointwise convergence mentioned above. Moreover, a slight modification of the argument based on (56) shows that, for any given continuous function ψ, the convergence (59) is uniform with respect to t∈[a,b] over any compact time interval $\left[a,b\right]\subset R_{+}$ not containing 0 (that is, 0<a⩽b). This is due to the uniform continuity and boundedness of ψ on [a,b] in combination with the fact that the properties (58) are satisfied for (57) uniformly over such an interval.

Although the processes $V_{T}$, $W_{T}$, $Z_{T}$ in (22)–(24) inherit nonsmoothness of sample paths from *V*, *W*, *Z*, they capture (approximately) only relatively slow components of the original processes (with frequencies ≪Ω or, equivalently, time scales ≫T). Since practical applications are usually concerned with the system behaviour in a bounded frequency range (specified by the cutoff frequency Ω), we will describe the sensitivity of the system output to the input noise in terms of the filtered processes by taking advantage of the smoothing effect of their ODE dynamics (51)–(53).

## 5. Root-Mean-Square Gain for Filtered Processes

For a given value of the time scale parameter T>0, the RMS gain of the system *F* under consideration can be quantified in terms of the filtered input and output $W_{T}$, $Z_{T}$ in (23) and (24) by

(60) $$\left[\;\right]F\left[\;\right]:=\underset{t\to +\infty }{\mathrm{lim sup}}\sqrt{\frac{\int_{0}^{t}E(|Z_{T}\left(\tau \right){|}^{2})d\tau }{\int_{0}^{t}E(|W_{T}\left(\tau \right){|}^{2})d\tau }}$$

(the trivial case of zero inputs is not considered). The following theorem, which is a continuous-time counterpart of [12] [Lemma 2], shows that the upper limit in (60) is in fact a limit. Its formulation employs an auxiliary function

(61) $$\Sigma \left(\omega \right):={\left|\Phi \left(i\omega \right)\right|}^{2}=\frac{2\Omega }{\Omega^{2}+\omega^{2}}=\frac{2T}{1+{\left(\omega T\right)}^{2}},\;\omega \in R,$$

which is associated with (50). The scalar function Σ specifies the spectral density $\Sigma I_{m}$ of the OU process $V_{T}$ in $R^{m}$ from (22) as the Fourier transform of the covariance function (26) in the stationary regime:

(62) $$\Sigma \left(\omega \right)=\int_{R}e^{-i\omega t-\Omega |t|}dt.$$

In a similar fashion, the functions Σ(ω)S(ω) and $\Sigma \left(\omega \right)F\left(i\omega \right)S\left(\omega \right)F{\left(i\omega \right)}^{*}$, with ω∈R, describe the spectral densities of the filtered noise and output processes $W_{T}$, $Z_{T}$ in (23) and (24), respectively, where *S* is the spectral density (40) for the increments of the process *W* in the sense of (3) and (41).

**Theorem** **1.** *Suppose the system F in (7) and (11) and the noise shaping filter G in (15) are stable. Then the RMS gain (60) holds as a limit and is computed as*

(63) $$\left[\;\right]F\left[\;\right]=\sqrt{\frac{\lim_{t\to +\infty }E(|Z_{T}\left(t\right){|}^{2})}{\lim_{t\to +\infty }E(|W_{T}\left(t\right){|}^{2})}}=\sqrt{\frac{\int_{R}\Sigma \left(\omega \right)\langle \Lambda \left(\omega \right),S\left(\omega \right)\rangle d\omega }{\int_{R}\Sigma \left(\omega \right)\mathrm{Tr}S\left(\omega \right)d\omega }}.$$

*Here, the function* Σ *is given by (61), 〈·,·〉 is the Frobenius inner product of matrices, and the spectral density S of the process W and the function $\Lambda :R\to H_{m}^{+}$ are associated with the transfer functions (37)–(39) by (40) and*

(64) $$\begin{array}{cc} \Lambda (\omega )\; & :=F{\left(i\omega \right)}^{*}F\left(i\omega \right),\;\omega \in R. \end{array}$$

**Proof.**  In view of (15), the solution (29) of the SDE (23) with the zero initial condition from (27) takes the form

(65) $$\begin{array}{cc} W_{T}\left(t\right)\; & =\int_{0}^{t}\phi \left(t-\tau \right)\left(c\xi \left(\tau \right)d\tau +ddV\left(\tau \right)\right)\\ \; & =\int_{0}^{t}\phi \left(t-\tau \right)\left(c\int_{0}^{\tau }e^{(\tau -u)\alpha }\beta dV\left(du\right)d\tau +ddV\left(\tau \right)\right)\\ \; & =\int_{0}^{t}h\left(t-u\right)dV\left(u\right). \end{array}$$

Here,

(66) $$\begin{array}{cc} h(\tau )\; & :=\left(\phi *\psi \right)\left(\tau \right)=\int_{0}^{\tau }\phi \left(\tau -u\right)\psi \left(u\right)du\\ \; & =\int_{0}^{\tau }\phi \left(\tau -u\right)ce^{u\alpha }\beta du+\phi \left(\tau \right)d,\;\tau ⩾0, \end{array}$$

is the convolution of the impulse responses of the low-pass filter Φ and the noise shaping filter *G*, which are given by (31) and

$$\psi \left(\tau \right):=ce^{\tau \alpha }\beta +d\delta \left(\tau \right),\;\tau ⩾0,$$

with δ the Dirac delta function [59]. Since *V* is a standard Wiener process in $R^{m}$, application of the Ito isometry to (65) leads to

(67) $$E(|W_{T}{\left(t\right)|}^{2})=\int_{0}^{t}{∥h\left(u\right)∥}_{F}^{2}du,\;t⩾0.$$

Due to the square integrability of the transfer function ΦG (which is the Laplace transform of the function $h:R_{+}\to R^{m\times m}$ from (66)) over the imaginary axis combined with the Plancherel theorem, the function *h* is square integrable over $R_{+}$, and (67) leads to the monotonic convergence

$$\begin{array}{cc} \lim_{t\to +\infty }E(|W_{T}\left(t\right){|}^{2})\; & =\int_{R_{+}}{∥h\left(u\right)∥}_{F}^{2}du\\ \; & =\frac{1}{2\pi }\int_{R}{\left|\Phi \left(i\omega \right)\right|}^{2}{∥G\left(i\omega \right)∥}_{F}^{2}d\omega \\ \; & =\frac{1}{2\pi }\int_{R}\Sigma \left(\omega \right)\mathrm{Tr}S\left(\omega \right)d\omega \end{array}$$

in view of (61) and (40). The same limit is shared by the Cesaro means of $E(|W_{T}\left(\tau \right){|}^{2})$ as a function of τ⩾0:

(68) $$\begin{array}{cc} \lim_{t\to +\infty }\left(\frac{1}{t}\int_{0}^{t}E(|W_{T}\left(\tau \right){|}^{2})d\tau \right)\; & =\lim_{t\to +\infty }E(|W_{T}\left(t\right){|}^{2})\\ \; & =\frac{1}{2\pi }\int_{R}\Sigma \left(\omega \right)\mathrm{Tr}S\left(\omega \right)d\omega . \end{array}$$

By applying a similar reasoning to the filtered system output $Z_{T}$ governed by (24), it follows that

(69) $$\begin{array}{cc} \lim_{t\to +\infty }\left(\frac{1}{t}\int_{0}^{t}E(|Z_{T}\left(\tau \right){|}^{2})d\tau \right)\; & =\lim_{t\to +\infty }E(|Z_{T}\left(t\right){|}^{2})\\ \; & =\frac{1}{2\pi }\int_{R}\Sigma \left(\omega \right){∥F\left(i\omega \right)G\left(i\omega \right)∥}_{F}^{2}d\omega \\ \; & =\frac{1}{2\pi }\int_{R}\Sigma \left(\omega \right)\mathrm{Tr}\left(F\left(i\omega \right)S\left(\omega \right)F{\left(i\omega \right)}^{*}\right)d\omega \\ \; & =\frac{1}{2\pi }\int_{R}\Sigma \left(\omega \right)\langle \Lambda \left(\omega \right),S\left(\omega \right)\rangle d\omega , \end{array}$$

where (64) is also used. A combination of (68) and (69) implies the existence of a limit in (60) which is given by (63). □

The RMS gain []F[] in (63) is a semi-norm of the system transfer function *F* in (37). It is well-defined for any bounded (and not necessarily integrable) spectral density *S* of the noise (the trivial case when S=0 almost everywhere is not considered). This is secured by the presence of the integrable function Σ from (61) as a factor in the integrands. The dependence of the RMS gain on the time scale parameter T>0 and the noise spectral density *S* will be indicated by the subscripts as

$$\left[\;\right]F\left[\;\right]=\left[\;\right]F{\left[\;\right]}_{T,S}.$$

Note that $\left[\;\right]F{\left[\;\right]}_{T,S}$ is invariant under the scaling S↦κS with an arbitrary constant κ>0 (which cancels out in the numerator and denominator in (63)):

(70) $$\left[\;\right]F{\left[\;\right]}_{T,\kappa S}=\left[\;\right]F{\left[\;\right]}_{T,S}.$$

Also, if the system *F* is isometric (that is, inner) up to a nonzero multiplicative constant, so that there exists λ>0 such that the function (64) satisfies $\Lambda \left(\omega \right)=\lambda I_{m}$ for all ω∈R, then the RMS gain reduces to

(71) $$\left[\;\right]F{\left[\;\right]}_{T,S}=\sqrt{\frac{\int_{R}\Sigma \left(\omega \right)\langle \lambda I_{m},S\left(\omega \right)\rangle d\omega }{\int_{R}\Sigma \left(\omega \right)\mathrm{Tr}S\left(\omega \right)d\omega }}=\sqrt{\lambda }$$

and does not depend on the noise spectral density *S*. In what follows, such systems *F* will be referred to as *round* systems (with all the other systems being called *nonround*). In general, application of the inequalities

$$\lambda_{\min}\left(L\right)\mathrm{Tr}M⩽\langle L,M\rangle ⩽\lambda_{\max}\left(L\right)\mathrm{Tr}M,$$

which hold for arbitrary Hermitian matrices *L*, *M* of equal dimensions with M≽0, leads to

(72) $$\underset{\omega \in R}{\inf}\sqrt{\lambda_{\min}\left(\Lambda \left(\omega \right)\right)}⩽\left[\;\right]F{\left[\;\right]}_{T,S}⩽\underset{\omega \in R}{\sup}\sqrt{\lambda_{\max}\left(\Lambda \left(\omega \right)\right)}={∥F∥}_{\infty }.$$

Here, $\lambda_{\min}\left(\cdot \right)$, $\lambda_{\max}\left(\cdot \right)$ are the smallest and largest eigenvalues of a Hermitian matrix, and ${∥\cdot ∥}_{\infty }$ is the $H_{\infty }$-norm [1] of a transfer function, so that (71) is a particular case of (72). If the input and output dimensions of the system satisfy m>p, then the matrix Λ(ω) in (64) is singular (at every frequency ω∈R), thus making the left-hand side of (72) vanish. For arbitrary dimensions, the RMS gain $\left[\;\right]F{\left[\;\right]}_{T,S}$ can approach any intermediate value in (72) by an appropriate choice of the spectral density *S* as discussed below.

**Theorem** **2.** *Suppose the system F in (7) and (11) is stable. Then for any μ from the interval in (72), that is, satisfying*

$$\underset{\omega \in R}{\inf}\sqrt{\lambda_{\min}\left(\Lambda \left(\omega \right)\right)}⩽\mu ⩽{∥F∥}_{\infty },$$

*and any ϵ>0, there exists a rational spectral density S (associated with a stable noise shaping filter G in (15)) such that*

$$|\left[\;\right]F{\left[\;\right]}_{T,S}-\mu |<\epsilon .$$

**Proof.**  With any γ>0, a frequency $\omega_{0}\in R$ and a unit vector $u\in C^{m}$, we associate a rational spectral density $S_{\gamma }:R\to H_{m}^{+}$:

(73) $$S_{\gamma }\left(\omega \right):=\frac{\gamma }{\pi }\left(\frac{1}{\gamma^{2}+{\left(\omega -\omega_{0}\right)}^{2}}uu^{*}+\frac{1}{\gamma^{2}+{\left(\omega +\omega_{0}\right)}^{2}}\bar{u}u^{T}\right),$$

which satisfies $\int_{R}\mathrm{Tr}S_{\gamma }\left(\omega \right)d\omega =2$. Due to the weak convergence of the Cauchy distribution with the PDF

$$\frac{1}{\pi }\frac{\gamma }{\gamma^{2}+{\left(\omega -\omega_{0}\right)}^{2}},\;\omega \in R,$$

to the atomic probability measure concentrated at $\omega_{0}$, as γ→0+ (and similarly for the opposite frequency $-\omega_{0}$), the spectral density (73) is convergent in the distributional sense [59] as

(74) $$\lim_{\gamma \to 0+}S_{\gamma }\left(\omega \right)=\delta \left(\omega -\omega_{0}\right)uu^{*}+\delta \left(\omega +\omega_{0}\right)\bar{u}u^{T}.$$

Since the functions Σ, Λ in (61) and (64) are bounded and continuous, then (74) implies that

(75) $$\begin{array}{cc} \lim_{\gamma \to 0+}\int_{R}\Sigma \left(\omega \right)\langle \Lambda \left(\omega \right),S_{\gamma }\left(\omega \right)\rangle d\omega \; & =2\Sigma \left(\omega_{0}\right){∥u∥}_{\Lambda (\omega_{0})}^{2}, \end{array}$$

(76) $$\begin{array}{cc} \lim_{\gamma \to 0+}\int_{R}\Sigma \left(\omega \right)\mathrm{Tr}S_{\gamma }\left(\omega \right)d\omega \; & =2\Sigma (\omega_{0}), \end{array}$$

where ${∥v∥}_{M}:=\left|\sqrt{M}v\right|=\sqrt{v^{*}Mv}$ is a weighted Euclidean semi-norm of a complex vector *v* specified by an appropriately dimensioned positive semi-definite Hermitian matrix *M*. Here, use is also made of the assumption |u|=1 and the property

(77) $$\Lambda \left(-\omega \right)=\bar{\Lambda (\omega )}=\Lambda {\left(\omega \right)}^{T},$$

whereby Λ(−ω) is isospectral to Λ(ω), and ${∥\bar{u}∥}_{\Lambda (-\omega )}={∥u∥}_{\Lambda (\omega )}$. In view of (75) and (76), the corresponding RMS gain in (63) satisfies

(78) $$\lim_{\gamma \to 0+}\left[\;\right]F{\left[\;\right]}_{T,S_{\gamma }}={∥u∥}_{\Lambda (\omega_{0})}\in \theta \left(\omega_{0}\right),$$

where θ is an auxiliary set-valued function which maps a frequency ω∈R to the interval

(79) $$\theta \left(\omega \right):=\left[\sqrt{\lambda_{\min}\left(\Lambda \left(\omega \right)\right)},\;\sqrt{\lambda_{\max}\left(\Lambda \left(\omega \right)\right)}\right].$$

Any given point of $\theta (\omega_{0})$ in (78) is achievable by an appropriate choice of a unit vector $u\in C^{m}$ since

$$\left\{{∥u∥}_{\Lambda (\omega )}:\;u\in C^{m},\;\left|u\right|=1\right\}=\theta \left(\omega \right)$$

for any ω∈R. With the endpoints of this interval being continuous even functions of ω (the continuity is inherited from Λ), the map θ in (79) covers the interior of the interval in (72) in the sense that

$$\left(\underset{\omega \in R}{\inf}\sqrt{\lambda_{\min}\left(\Lambda \left(\omega \right)\right)},\;{∥F∥}_{\infty }\right)\subset \underset{\omega \in R}{⋃}\theta \left(\omega \right).$$

Therefore, for any μ from the interval in (72) and any ϵ>0, there exists $\omega_{0}\in R$ and a unit vector $u\in C^{m}$ such that $|{∥u∥}_{\Lambda (\omega_{0})}-\mu |<\epsilon$. In view of (78), the corresponding spectral density $S_{\gamma }$ in (73), associated with such $\omega_{0}$ and *u*, delivers the RMS gain which satisfies $|\left[\;\right]F{\left[\;\right]}_{T,S_{\gamma }}-{∥u∥}_{\Lambda (\omega_{0})}|\;<\epsilon$ for all sufficiently small γ>0, and hence, $|\left[\;\right]F{\left[\;\right]}_{T,S_{\gamma }}-\mu |<2\epsilon$ by the triangle inequality. □

It follows from Theorem 2 that, in the absence of specific additional constraints on the spectral density *S* of the noise *W*, the second inequality in (72) cannot be improved in the sense that

$$\underset{S}{\sup}\left[\;\right]F{\left[\;\right]}_{T,S}={∥F∥}_{\infty }.$$

Moreover, the supremum can be restricted (without affecting its value) to the class of rational spectral densities $S:R\to H_{m}^{+}$. Indeed, the proof of the theorem provides a particular way to construct a maximizing sequence of rational spectral densities $S_{\gamma }$ in (73), with *u* being a unit eigenvector of the matrix $\Lambda (\omega_{0})$ associated with its largest eigenvalue. Such spectral densities exhibit “energy concentration” about certain frequencies and in certain directions in $C^{m}$, which depend on the system *F* and, being exceptional in this sense, are not necessarily “targeted” by the noise *W*, especially when the latter, up to scaling, is not far from the isotropic standard Wiener process.

## 6. RMS Gain with Respect to Isotropic White-Noise Inputs

As opposed to the energy concentration used in the proof of Theorem 2, consider an isotropic white-noise case when the spectral density of the input *W* is a constant scalar matrix:

(80) $$S\left(\omega \right)=\kappa I_{m},\;\omega \in R,$$

where κ>0 is a scalar parameter. In this case, *W* is a standard Wiener process up to the multiplicative constant $\sqrt{\kappa }$ (and can be obtained from *V* by letting g=0, $g_{0}=\sqrt{\kappa }I_{m}$ in (14)), so that the SDE (23) in its stationary regime makes $\frac{1}{\sqrt{\kappa }}W_{T}$ an OU process. Then, by substituting (80) into (63), the RMS gain takes the form

(81) $$\begin{array}{cc} \left[\;\right]F{\left[\;\right]}_{T,\kappa I_{m}}\; & =\left[\;\right]F{\left[\;\right]}_{T,I_{m}}=\sqrt{\frac{\int_{R}\Sigma \left(\omega \right)\mathrm{Tr}\Lambda \left(\omega \right)d\omega }{m\int_{R}\Sigma \left(\omega \right)d\omega }}\\ \; & =\sqrt{\frac{1}{2\pi m}\int_{R}\Sigma \left(\omega \right)\mathrm{Tr}\Lambda \left(\omega \right)d\omega }\\ \; & =\sqrt{\frac{1}{2\pi m}\int_{T}\mathrm{Tr}\Lambda \left(\Omega \tan\frac{\varphi }{2}\right)d\varphi }, \end{array}$$

since the function Σ in (61) satisfies

(82) $$\int_{R}\Sigma \left(\omega \right)d\omega =2\pi ,$$

in accordance with the Fourier transform (62). In (81), use is also made of the frequency transformation

(83) $$\omega =\Omega \tan\frac{\varphi }{2},\;\varphi :=2\arctan\left(\omega T\right),$$

so that

(84) $$\partial_{\omega }\varphi =\frac{2T}{1+{\left(\omega T\right)}^{2}}=\Sigma \left(\omega \right)$$

(or, equivalently, Σ(ω)dω=dφ), where the new integration variable φ takes values in the interval

(85) T:=(−π,π),

which represents the unit circle

$$\left\{e^{i\varphi }:\;\varphi \in T\right\}=\left\{z\in C:\;|z|=1\right\}\setminus \left\{-1\right\}$$

in the complex plane, punctured at −1. The right-hand side of (81) does not depend on κ in accordance with the scale invariance (70) and is organized as a weighted $H_{2}$-norm [1] of the transfer function *F*, which involves the parameter Ω from (25).

We will now discuss the asymptotic behaviour of the RMS gain (81) as a function of *T*, when T→+∞ and T→0+.

As T→+∞ (so that Ω→0+), the OU process $W_{T}$ acquires long-range correlations. In this case, the spectral density Σ in (61) converges to 2πδ in the distributional sense. The effect of such a process on the system is, in essence, equivalent to that of a constant input. The corresponding limit

(86) $$\lim_{T\to +\infty }\left[\;\right]F{\left[\;\right]}_{T,I_{m}}=\lim_{\Omega \to 0+}\left[\;\right]F{\left[\;\right]}_{T,I_{m}}=\sqrt{\frac{1}{m}\mathrm{Tr}\Lambda \left(0\right)}=\frac{1}{\sqrt{m}}{∥F\left(0\right)∥}_{F},$$

which is obtained by applying Lebesgue’s dominated convergence theorem to the integral in the RMS gain (81), involves the static gain matrix $F\left(0\right)=D-CA^{-1}B$ of the system in view of (37).

By a similar reasoning, as the correlation time *T* of the OU input $W_{T}$ goes to zero (or, equivalently, as Ω→+∞), the spectral density (61) becomes constant (Σ(ω)≈2T) over the widening frequency interval |ω|≪Ω, in which case, the RMS gain (81) approaches a different limit:

(87) $$\lim_{T\to 0+}\left[\;\right]F{\left[\;\right]}_{T,I_{m}}=\lim_{\Omega \to +\infty }\left[\;\right]F{\left[\;\right]}_{T,I_{m}}=\sqrt{\frac{1}{m}\mathrm{Tr}\lim_{\omega \to \infty }\Lambda \left(\omega \right)}=\frac{1}{\sqrt{m}}{∥D∥}_{F}.$$

The limits (86) and (87) of the RMS gain (with respect to an isotropic white noise in the sense of (80)) do not reduce to the standard $H_{2}$-norm

(88) $${∥F∥}_{2}:=\sqrt{\frac{1}{2\pi }\int_{R}\mathrm{Tr}\Lambda \left(\omega \right)d\omega }$$

which is finite only when the system transfer function (37) is strictly proper, that is, if D=0. In the latter case, the limit in (87) vanishes, and the asymptotic behaviour of the RMS gain is described by

$$\left[\;\right]F{\left[\;\right]}_{T,I_{m}}=\sqrt{\frac{T}{\pi m}\int_{R}\frac{\mathrm{Tr}\Lambda (\omega )}{1+{\left(\omega T\right)}^{2}}d\omega }∼{∥F∥}_{2}\sqrt{\frac{2T}{m}},\;\mathrm{as}\;T\to 0+,$$

where use is made of the last equality in (61) in combination with the limit relation

$$\lim_{T\to 0}\int_{R}\frac{\mathrm{Tr}\Lambda (\omega )}{1+{\left(\omega T\right)}^{2}}d\omega =2\pi {∥F∥}_{2}^{2},$$

which follows from (88) by Lebesgue’s dominated convergence theorem since TrΛ(ω) is integrable over ω∈R for strictly proper stable systems *F*.

The limits (86) and (87) in the isotropic white-noise case being discussed manifest themselves when the time scale parameter *T* of the low-pass filtering is large, or, respectively, small, in comparison with the transient times in the system *F*, which can be described in terms of the set

$$T:=\left\{\frac{1}{\left|\lambda \right|}:\;\lambda \in S\right\},$$

where S denotes the spectrum of the Hurwitz matrix *A*. The fulfillment of either of the relations

(89) $$T\ll \min T=\frac{1}{r(A)},\;\max T=r\left(A^{-1}\right)\ll T$$

(with r(·) the spectral radius of a square matrix) indicates whether the OU process $W_{T}$ is nearly white or strongly coloured for the system *F*, respectively. The relative simplicity of this comparison comes from the fact that the OU process has only one time scale parameter *T* (which specifies the characteristic width Ω of the effective frequency range for the process), whereas more complicated random inputs can have multiple time scales, similarly to the system *F* itself.

The behaviour of the RMS gain with respect to isotropic white-noise inputs, considered above, is qualitatively different from its discrete-time counterpart [12] despite similarities in their definitions. Indeed, in the discrete-time settings, the frequency range is finite and can be identified with the interval T in (85), whereas the continuous-time processes may contain arbitrarily fast components (with arbitrarily short time scales or, equivalently, high frequencies) and have the infinite frequency range.

On the other hand, the last representation in (81) is in terms of the $H_{2}$-norm of a discrete-time transfer function related to (37) by a conformal correspondence. This correspondence applies not only to the isotropic white-noise case but also to a wide class of stationary Gaussian disturbances discussed below.

## 7. Conformal Correspondence Between Continuous and Discrete Time Settings

For what follows, consider an involutive conformal map (a modified Cayley transform [50])

(90) $$K\left(z\right)=\frac{1-z}{1+z}$$

between the open unit disk and the open right half-plane in the complex plane:

(91) K:{z∈C:|z|<1}→{s∈C:Res>0}.

These two domains pertain to the discrete and continuous time settings, respectively. The map *K* is a smooth bijection of the punctured unit circle {z∈C:|z|=1}∖{−1} onto the imaginary axis:

(92) $$K\left(e^{i\varphi }\right)=\frac{e^{-i\varphi /2}-e^{i\varphi /2}}{e^{-i\varphi /2}+e^{i\varphi /2}}=-i\tan\frac{\varphi }{2},\;\varphi \in T,$$

with T from (85). This property is inherited by the scaled version ΩK of the map *K* for any cutoff frequency Ω>0 in (25). The resulting conformal map ΩK gives rise to a linear operator Ψ, which is associated with the given time scale parameter *T* and maps the transfer functions *F*, *G* of the system and the noise shaping filter in (37)–(39) to the transfer functions

(93) $$\begin{array}{cc} F_{T}\left(z\right) & :=\Psi (F)(z):=F(\Omega K(z)), \end{array}$$

(94) $$\begin{array}{cc} G_{T}\left(z\right) & :=\Psi (G)(z):=G(\Omega K(z)), \end{array}$$

which are analytic in the open unit disk {z∈C:|z|<1} and correspond to stable LDTI systems. Up to a factor of two in ΩK, the operator Ψ is identical to Tustin’s transform [51] which converts LCTI systems to LDTI systems and vice versa. Similarly to (64) and (40), we associate with $F_{T}$, $G_{T}$ the functions $\Lambda_{T},S_{T}:T\to H_{m}^{+}$ by

(95) $$\begin{array}{cc} \Lambda_{T}\left(\varphi \right)\; & :=F_{T}{\left(e^{i\varphi }\right)}^{*}F_{T}\left(e^{i\varphi }\right)=\Lambda \left(-\Omega \tan\frac{\varphi }{2}\right), \end{array}$$

(96) $$\begin{array}{cc} S_{T}\left(\varphi \right)\; & :=G_{T}\left(e^{i\varphi }\right)G_{T}{\left(e^{i\varphi }\right)}^{*}=S\left(-\Omega \tan\frac{\varphi }{2}\right), \end{array}$$

where the rightmost equalities follow from (92). The significance of these functions is clarified below by using the connection (84) between the spectral density Σ of the OU process in (61) and the logarithmic derivative

$${\left(\ln K\left(s\right)\right)}^{\prime }=\frac{2}{s^{2}-1}$$

of the conformal map *K* in (90) (recall that, being an involution, *K* coincides with its functional inverse $K^{-1}$).

**Theorem** **3.** *Suppose the system F in (7) and (11) and the noise shaping filter G in (15) are stable. Then the RMS gain (63) can be represented in terms of the functions $\Lambda_{T}$, $S_{T}$ from (95) and (96) as*

(97) $$\begin{array}{cc} \left[\;\right]F{\left[\;\right]}_{T,S}\; & =\sqrt{\frac{\int_{T}\langle \Lambda_{T}\left(\varphi \right),S_{T}\left(\varphi \right)\rangle d\varphi }{\int_{T}\mathrm{Tr}S_{T}\left(\varphi \right)d\varphi }}. \end{array}$$

**Proof.**  Similarly to (77), the spectral density *S* in (40) satisfies $S\left(-\omega \right)=\bar{S(\omega )}=S{\left(\omega \right)}^{T}$, and hence, both 〈Λ(ω),S(ω)〉=〈Λ(−ω),S(−ω)〉 and TrS(ω)=TrS(−ω) are even functions of ω∈R. In combination with the frequency transformation in (83) and (84), this allows the numerator and denominator in (63) to be represented as

(98) $$\begin{array}{cc} \int_{R}\Sigma \left(\omega \right)\langle \Lambda \left(\omega \right),S\left(\omega \right)\rangle d\omega \; & =\int_{R}\Sigma \left(\omega \right)\langle \Lambda \left(-\omega \right),S\left(-\omega \right)\rangle d\omega \\ \; & =\int_{T}\langle \Lambda (-\Omega \tan\frac{\varphi }{2}),S(-\Omega \tan\frac{\varphi }{2})\rangle d\varphi \\ \; & =\int_{T}\langle \Lambda_{T}\left(\varphi \right),S_{T}\left(\varphi \right)\rangle d\varphi , \end{array}$$

(99) $$\begin{array}{cc} \int_{R}\Sigma \left(\omega \right)\mathrm{Tr}S\left(\omega \right)d\omega \; & =\int_{R}\Sigma \left(\omega \right)\mathrm{Tr}S\left(-\omega \right)d\omega \\ \; & =\int_{T}\mathrm{Tr}S(-\Omega \tan\frac{\varphi }{2})d\varphi \\ \; & =\int_{T}\mathrm{Tr}S_{T}\left(\varphi \right)d\varphi , \end{array}$$

where the integration variable change is used along with (95) and (96). Substitution of (98) and (99) into (63) establishes (97). □

Note that the function $S_{T}:T\to H_{m}^{+}$ in (96) is the spectral density of an auxiliary stationary zero-mean Gaussian random sequence

(100) $$\varpi :={\left(\varpi_{k}\right)}_{k\in Z}$$

in $R^{m}$ whose elements are indexed by the set of integers Z. Their variance (which is constant in time due to stationarity) is

(101) $$\begin{array}{cc} E(|\varpi_{k}{|}^{2})\; & =\frac{1}{2\pi }\int_{T}\mathrm{Tr}S_{T}\left(\varphi \right)d\varphi \\ \; & =\frac{1}{2\pi }\int_{R}\Sigma \left(\omega \right)\mathrm{Tr}S\left(\omega \right)d\omega ,\;k\in Z. \end{array}$$

**Figure 4:**
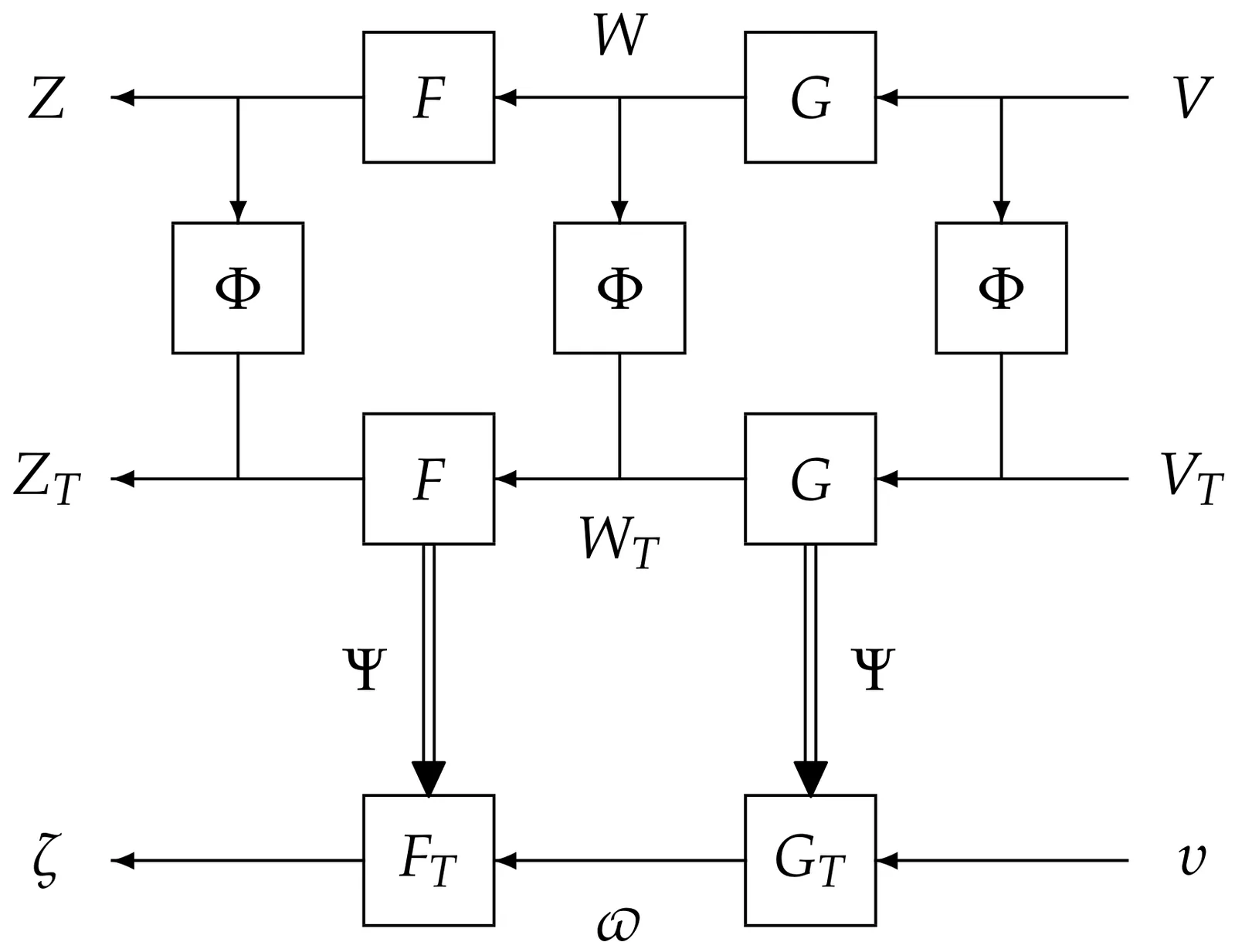
The block diagram of Figure 3 augmented by the linear operator Ψ (represented by double arrows) which maps the continuous-time transfer functions *F*, *G* to their discrete-time counterparts $F_{T}$, $G_{T}$ in (93) and (94). Also shown are the auxiliary random sequences ζ, ϖ, υ related by the LDTI systems $F_{T}$, $G_{T}$. The RMS gain of *F* with respect to $W_{T}$ in (63) is equal to that of $F_{T}$ with respect to ϖ in (106).

The sequence ϖ is the output of an LDTI shaping filter (with the transfer function $G_{T}$ in (94)) driven by a Gaussian white-noise sequence

(102) $$\upsilon :={\left(\upsilon_{k}\right)}_{k\in Z}$$

which consists of mutually independent standard normal random vectors in $R^{m}$, so that

(103) $$E\upsilon_{k}=0,\;E\left(\upsilon_{j}\upsilon_{k}^{T}\right)=\delta_{jk}I_{m},\;j,k\in Z,$$

where $\delta_{jk}$ is the Kronecker delta. At the same time, ϖ from (100) is the input to an LDTI system (with the transfer function $F_{T}$ in (93)) whose output is a stationary zero-mean Gaussian random sequence

(104) $$\zeta :={\left(\zeta_{k}\right)}_{k\in Z}$$

in $R^{p}$. The resulting setup is shown in Figure 4.

Since the spectral density of the sequence ζ is given by $F_{T}\left(e^{i\varphi }\right)S_{T}\left(\varphi \right)F_{T}{\left(e^{i\varphi }\right)}^{*}$, the common variance of its elements is computed as

(105) $$\begin{array}{cc} E(|\zeta_{k}{|}^{2})\; & =\frac{1}{2\pi }\int_{T}\mathrm{Tr}\left(F_{T}\left(e^{i\varphi }\right)S_{T}\left(\varphi \right)F_{T}{\left(e^{i\varphi }\right)}^{*}\right)d\varphi \\ \; & =\frac{1}{2\pi }\int_{T}\langle \Lambda_{T}\left(\varphi \right),S_{T}\left(\varphi \right)\rangle d\varphi \\ \; & =\frac{1}{2\pi }\int_{R}\Sigma \left(\omega \right)\langle \Lambda \left(\omega \right),S\left(\omega \right)\rangle d\omega ,\;k\in Z. \end{array}$$

In view of (101) and (105) (see also [12] [Lemma 2]), the RMS gain (97) of the system *F* is identical to that of its discrete-time counterpart $F_{T}$ specified above:

(106) $$\left[\;\right]F{\left[\;\right]}_{T,S}=\sqrt{\frac{E(|\zeta_{0}{|}^{2})}{E(|\varpi_{0}{|}^{2})}}=\left[\;\right]F_{T}{\left[\;\right]}_{S_{T}},$$

where, without loss of generality, the variances in the numerator and denominator are taken at the initial moment of time due to stationarity of the sequences (100) and (104). Note that in the stationary case, it is redundant to consider Cesaro means of moments (such as variances) instead of their constant values. The right-hand side of (106) is the RMS gain of the discrete-time transfer function $F_{T}$ with respect to stationary zero-mean Gaussian random sequences ϖ with the spectral density $S_{T}$. This quantity depends on the spectral density *S* of the continuous time process *W* through its discrete-time image $S_{T}$ in (96) and can be computed for a given *S* both in the continuous and discrete time domains due to the *T*-dependent conformal correspondence between them.

For example, if *W* is a standard Wiener process up to a multiplicative constant (the isotropic white-noise case (80)), then the spectral density $S_{T}$ in (96) is the same constant scalar matrix, and ϖ is a zero-mean Gaussian white-noise sequence with the scalar covariance matrix. In this case, (106) reproduces (81). However, the conformal correspondence applies to a broader setting of worst-case RMS gains with respect to statistically uncertain disturbances, where the uncertainty can be formulated in terms of the discrete-time counterparts of the system and the noise shaping filter.

## 8. Anisotropy-Constrained Statistical Uncertainty and Worst-Case RMS Gain

Suppose the input *W* of the system *F* is a statistically uncertain random disturbance, which is produced at the output of an imprecisely known noise shaping filter *G* in Figure 1, with the only prior information about its spectral density *S* being that it belongs to a given class S. Then of interest is the worst-case value

(107) $$\left[\;\right]F{\left[\;\right]}_{T,S}:=\underset{S\in S}{\sup}\left[\;\right]F{\left[\;\right]}_{T,S}$$

of the RMS gain (106) (we have slightly abused the notation). In particular, the class S can be described in terms of the deviation of *S* from constant scalar matrices (which correspond to the nominal isotropic white-noise model (80)). Such deviation is quantified, for example, by the mean anisotropy [12,18] of the stationary Gaussian random sequence (100) in $R^{m}$, which, in view of (95) and (101), takes the form

(108) $$\begin{array}{cc} A_{T}\left(S\right)\; & :=\bar{A}\left(S_{T}\right)=-\frac{1}{2}\ln\det\left(\frac{m}{E(|\varpi_{0}{|}^{2})}\Gamma \right)\\ \; & =-\frac{1}{4\pi }\int_{T}\ln\det\left(\frac{m}{E(|\varpi_{0}{|}^{2})}S_{T}\left(\varphi \right)\right)d\varphi \\ \; & =-\frac{1}{4\pi }\int_{R}\Sigma \left(\omega \right)\ln\det\left(\frac{2\pi m}{\int_{R}\Sigma \left(\lambda \right)\mathrm{Tr}S\left(\lambda \right)d\lambda }S\left(\omega \right)\right)d\omega \\ \; & =-\frac{T}{2\pi }\int_{R}\frac{1}{1+{\left(\omega T\right)}^{2}}\ln\det\left(\frac{\pi m}{T\int_{R}\frac{\mathrm{Tr}S(\lambda )}{1+{\left(\lambda T\right)}^{2}}d\lambda }S\left(\omega \right)\right)d\omega , \end{array}$$

where $0≺\Gamma =\Gamma^{T}\in R^{m\times m}$ is an auxiliary matrix which is discussed below along with the main properties of (108). The quantity $A_{T}\left(S\right)$ is finite and nonnegative if the spectral density *S* of the noise *W* satisfies the following inequality:

(109) $$\int_{T}\ln\det S_{T}\left(\varphi \right)d\varphi =2T\int_{R}\frac{\ln\det S(\omega )}{1+{\left(\omega T\right)}^{2}}d\omega >-\infty .$$

Although the fulfillment of this condition does not depend on a particular choice of T>0, an important role for the convergence of the second integral in (109) is played by the denominator. This is explained by the fact that an arbitrary spectral density $S:R\to H_{m}^{+}$ and its log-determinant cannot be simultaneously integrable on the real axis in view of the relations

(110) $$\int_{-b}^{b}\left(\mathrm{Tr}S\left(\omega \right)-\ln\det S\left(\omega \right)\right)d\omega ⩾2bm\to +\infty ,\;\mathrm{as}\;b\to +\infty ,$$

whereby $\int_{R}\mathrm{Tr}S\left(\omega \right)d\omega <+\infty$ implies that $\int_{R}\ln\det S\left(\omega \right)d\omega =-\infty$. The inequality (110) employs the following lower bound for the integrand:

(111) $$\min_{Q\in H_{m}^{+}}\left(\mathrm{Tr}Q-\ln\det Q\right)=m,$$

where the function being minimized is strictly convex and achieves its unique minimum at $Q=I_{m}$. This can be verified by using the Frechet derivatives $\partial_{Q}\mathrm{Tr}Q=I_{m}$ and $\partial_{Q}\ln\det Q=Q^{-1}$, with the latter assuming that Q≻0.

Since the transfer function $G_{T}$ in (94) belongs to the Hardy space of $C^{m\times m}$-valued functions, analytic in the open unit disk and having a square integrable boundary value over the unit circle, then (109) is equivalent to $\det G_{T}\left(\varphi \right)\neq 0$ for almost all φ∈T. This full-rank condition, associated with the inner-outer factorization [54] of the spectral density $S_{T}$ in (96), reflects the absence of linear dependencies between the entries of the random vectors $\varpi_{k}$ at the same or different moments of time. Also, this property is closely related to the total unpredictability [53,60] of such random sequences in the sense of nonsingularity of the conditional covariance matrix

(112) $$\Gamma :=\mathrm{cov}(\varpi_{k}|F_{k-1})$$

given the past history ${\left(\varpi_{j}\right)}_{j<k}$ of the sequence ϖ in (100). Here, $F:={\left(F_{k}\right)}_{k\in Z}$ is the natural filtration for ϖ consisting of the the σ-algebras

$$F_{k}:=\sigma \left\{\varpi_{k},\varpi_{k-1},\varpi_{k-2},\ldots \right\}.$$

In the stationary Gaussian case being considered, the matrix Γ (which is used in (108)) does not depend on discrete time *k* and is nonrandom, whereby it coincides with the unconditional covariance matrix of the one-step prediction error ${\tilde{\varpi }}_{k}$ associated with the sequence ϖ by

(113) $${\tilde{\varpi }}_{k}:=\varpi_{k}-{\hat{\varpi }}_{k},\;{\hat{\varpi }}_{k}:=E\left(\varpi_{k}|F_{k-1}\right).$$

The F-predictable sequence $\hat{\varpi }:={\left({\hat{\varpi }}_{k}\right)}_{k\in Z}$ and the F-martingale difference sequence $\tilde{\varpi }:={\left({\tilde{\varpi }}_{k}\right)}_{k\in Z}$ provide the Doob decomposition [61] for ϖ:

(114) $$\varpi_{k}={\hat{\varpi }}_{k}+{\tilde{\varpi }}_{k},\;k\in Z.$$

By the Szegö-Kolmogorov theorem [26,62], as discussed in the proof of [18] [Lemma 1], the matrix (112) is linked to the spectral density $S_{T}$ of ϖ as

(115) $$\ln\det\Gamma =\lim_{N\to +\infty }\frac{\ln\det\mathrm{cov}(\varpi^{N})}{N}=\frac{1}{2\pi }\int_{T}\ln\det S_{T}\left(\varphi \right)d\varphi ,$$

where

(116) $$\varpi^{N}:={\left(\varpi_{k}\right)}_{0⩽k<N}$$

is an $R^{mN}$-valued zero-mean Gaussian random vector formed from *N* consecutive elements $\varpi_{0},\ldots ,\varpi_{N-1}$ of the sequence ϖ in (100), whose covariance matrix is recovered from $S_{T}$ as

$$E\left(\varpi_{j}\varpi_{k}^{T}\right)=\frac{1}{2\pi }\int_{T}e^{i(j-k)\varphi }S_{T}\left(\varphi \right)d\varphi .$$

In application to the stationary zero-mean Gaussian sequence ϖ with the spectral density $S_{T}$, the origin of the mean anisotropy functional (108) is clarified by the limit theorems from [12] [Theorem 1] and [13] [p. 1269] as

$$\lim_{N\to +\infty }\frac{A_{\circ }\left({℧}_{N}\right)}{N}=\lim_{N\to +\infty }\frac{A(\varpi^{N})}{N}=\bar{A}\left(S_{T}\right).$$

Here, the original “spherical” anisotropy functional from [12]

(117) $$\begin{array}{cc} A_{\circ }\left({℧}_{N}\right)\; & :=D\left(Q_{N}∥U_{mN}\right)=E\ln q_{N}\left({℧}_{N}\right)\\ \; & =\int_{S_{mN}}q_{N}\left(u\right)\ln q_{N}\left(u\right)U_{mN}\left(du\right) \end{array}$$

is the KL relative entropy [11] of the distribution $Q_{N}$ of the unit random vector

$${℧}_{N}:=\frac{1}{|\varpi^{N}|}\varpi^{N}$$

with a PDF $q_{N}:S_{mN}\to R_{+}$ with respect to the uniform distribution $U_{mN}$ on the unit sphere

$$S_{mN}:=\{u\in R^{mN}:\;|u|=1\}.$$

The other anisotropy functional, introduced subsequently in [13] (and inspired by a similar yet one-dimensional power-entropy functional from [14]), is given by

(118) $$\begin{array}{cc} A(\varpi^{N})\; & :=\min_{\lambda >0}D\left(\Pi_{N}∥N_{\lambda I_{mN}}\right)\\ \; & =\frac{mN}{2}\ln\left(\frac{2\pi e}{mN}E(|\varpi^{N}{|}^{2})\right)-h\left(\varpi^{N}\right)\\ \; & =-\frac{1}{2}\ln\det\left(\frac{m}{E(|\varpi_{0}{|}^{2})}\mathrm{cov}\left(\varpi^{N}\right)\right), \end{array}$$

which is the minimum relative entropy of the distribution $\Pi_{N}$ of the random vector $\varpi^{N}$ from (116) with respect to isotropic Gaussian distributions $N_{\lambda I_{mN}}$ in $R^{mN}$ with zero mean and scalar covariance matrices $\lambda I_{mN}$. Here, h(·) is the differential entropy (with respect to the usual Lebesgue measure) which lends itself to closed-form computation for Gaussian random vectors, thus making the second anisotropy functional (118) more practical than its predecessor (117). Indeed,

(119) $$\begin{array}{cc} h(\varpi^{N})\; & :=-E\ln p_{N}\left(\varpi^{N}\right)\\ \; & =\frac{mN}{2}\ln\left(2\pi e\right)+\frac{1}{2}\ln\det\mathrm{cov}\left(\varpi^{N}\right), \end{array}$$

which is obtained by using the PDF $p_{N}:R^{mN}\to R_{+}$ of the random vector $\varpi^{N}$ from (116) given by

$$p_{N}\left(w\right)=\frac{{\left(2\pi \right)}^{-mN/2}}{\sqrt{\det\mathrm{cov}(\varpi^{N})}}e^{-\frac{1}{2}{∥w∥}_{\mathrm{cov}{\left(\varpi^{N}\right)}^{-1}}^{2}},\;w\in R^{mN}.$$

Note that (119) clarifies the entropy-theoretic meaning of (115) by its connection with the differential entropy rate

$$\lim_{N\to +\infty }\frac{h(\varpi^{N})}{N}=\frac{m}{2}\ln\left(2\pi e\right)+\frac{1}{2}\ln\det\Gamma =\frac{1}{2}\ln\det\left(2\pi e\Gamma \right)$$

as a particular case of the Shannon-McMillan-Breiman theorem [11] in application to the stationary Gaussian sequence ϖ.

The mean anisotropy $A_{T}\left(S\right)$ in (108) is invariant under the scaling and “rotation” transformations $S\left(\omega \right)\mapsto \lambda U\left(i\omega \right)S\left(\omega \right)U{\left(i\omega \right)}^{*}$ for any constant λ>0 and all-pass transfer functions *U* (that is, such that the matrix U(iω) is unitary at all frequencies ω∈R). Furthermore, $A_{T}\left(S\right)$, which is always nonnegative, vanishes if and only if the spectral density $S_{T}$ of the auxiliary sequence ϖ in (100), and hence, *S* in view of (96), is a constant scalar matrix, which (in the Gaussian case) holds if and only if the input disturbance *W* is a standard Wiener process up to a multiplicative constant. The above properties are inherited by $A_{T}\left(S\right)$ from its discrete-time counterpart, and so also is the closely related decomposition

(120) $$A_{T}\left(S\right)=A_{T}^{\mathrm{spat}}\left(S\right)+A_{T}^{\mathrm{temp}}\left(S\right)$$

of the mean anisotropy functional (108) into the “spatial” and “temporal” components (cf. [21] [Equations (7)–(9)])

(121) $$\begin{array}{cc} A_{T}^{\mathrm{spat}}\left(S\right)\; & :=-\frac{1}{2}\ln\det\left(\frac{m}{E(|\varpi_{0}{|}^{2})}E\left(\varpi_{0}\varpi_{0}^{T}\right)\right)\\ \; & =-\frac{1}{2}\ln\det\left(\frac{m}{\int_{R}\frac{\mathrm{Tr}S(\lambda )}{1+{\left(\lambda T\right)}^{2}}d\lambda }\int_{R}\frac{1}{1+{\left(\omega T\right)}^{2}}S\left(\omega \right)d\omega \right), \end{array}$$

(122) $$\begin{array}{cc} A_{T}^{\mathrm{temp}}\left(S\right)\; & :=\frac{1}{2}\ln\det\left(\Gamma^{-1}E\left(\varpi_{0}\varpi_{0}^{T}\right)\right)\\ \; & =\frac{T}{2\pi }\int_{R}\frac{1}{1+{\left(\omega T\right)}^{2}}\ln\det\left(\frac{T}{\pi }S{\left(\omega \right)}^{-1}\int_{R}\frac{1}{1+{\left(\lambda T\right)}^{2}}S\left(\lambda \right)d\lambda \right)d\omega , \end{array}$$

which are also nonnegative. In particular, $A_{T}^{\mathrm{temp}}\left(S\right)⩾0$ because this quantity coincides with Shannon’s mutual information [11] between an element $\varpi_{k}$ of the stationary Gaussian sequence ϖ and its past history ${\left(\varpi_{j}\right)}_{j<k}$:

$$\begin{array}{cc} A_{T}^{\mathrm{temp}}\left(S\right)\; & =I\left(\varpi_{k};{\left(\varpi_{j}\right)}_{j<k}\right)=h\left(\varpi_{k}\right)-h\left(\varpi_{k}|{\left(\varpi_{j}\right)}_{j<k}\right)\\ \; & =\lim_{N\to +\infty }I\left(\varpi_{0};{\left(\varpi_{-j}\right)}_{1⩽j⩽N}\right), \end{array}$$

where h(·∣·) is the conditional differential entropy. The nonnegativeness of $A_{T}^{\mathrm{temp}}\left(S\right)$ can also be verified directly by using the inequality $\Gamma ≼E(\varpi_{0}\varpi_{0}^{T})$ for the matrix (112) in the Gaussian case or, alternatively, Jensen’s inequality in combination with the normalization property of Σ in (82) and the strict concavity of lndet(·) on the cone of complex positive definite Hermitian matrices (which was used in (111)). The last argument also shows that $A_{T}^{\mathrm{temp}}\left(S\right)=0$ in (122) if and only if the spectral density *S* is a constant positive definite matrix, that is, when the noise process *W* is temporally white in the sense of having independent increments.

Similarly to discrete-time settings, the properties of $A_{T}\left(S\right)$ in (108) allow it to be used as a measure of deviation from the nominal isotropic white-noise model for specifying a particular uncertainty class S in (107):

(123) $$S_{T,a}:=\left\{S:R\to H_{m}^{+}:\;A_{T}\left(S\right)⩽a\right\}.$$

This class consists of all those spectral densities *S* of the input disturbance process *W*, whose discrete-time counterparts $S_{T}$ in (96) satisfy the upper constraint on the mean anisotropy (108) of the corresponding stationary Gaussian sequence ϖ with the variance (101). Since the map $S\mapsto S_{T}$ is bijective, the worst-case RMS gain (107), associated with (123), takes the form

(124) $$\begin{array}{cc} \left||\right|F{\left||\right|}_{T,a}\; & :=\left[\;\right]F{\left[\;\right]}_{T,S_{T,a}}=\underset{S\in S_{T,a}}{\sup}\left[\;\right]F{\left[\;\right]}_{T,S}\\ \; & =\underset{S\in S_{T,a}}{\sup}\sqrt{\frac{\int_{T}\langle \Lambda_{T}\left(\varphi \right),S_{T}\left(\varphi \right)\rangle d\varphi }{\int_{T}\mathrm{Tr}S_{T}\left(\varphi \right)d\varphi }}=\left||\right|F_{T}{\left||\right|}_{a} \end{array}$$

and coincides with the *a*-anisotropic norm of the discrete-time system $F_{T}$. The latter norm (and hence, the two-parameter (T,a)-anisotropic norm $\left||\right|F{\left||\right|}_{T,a}$ of the underlying LCTI system) lends itself to state-space computation [18].

## 9. Computation of Continuous-Time Anisotropic Norm in State Space

In order to compute the norm $\left||\right|F{\left||\right|}_{T,a}$ of the LCTI system *F* in state space through its discrete-time counterpart $\left||\right|F_{T}{\left||\right|}_{a}$ in (124), we note that, in view of (25), (37) and (90), the transfer function $F_{T}$ in (93) takes the form

(125) $$\begin{array}{cc} F_{T}\left(z\right) & =C{\left(\Omega \frac{1-z}{1+z}I_{n}-A\right)}^{-1}B+D\\ \; & =\left(1+z\right)TC{\left(I_{n}-TA-z\left(I_{n}+TA\right)\right)}^{-1}B+D\\ \; & =\left(1+z\right)C{\left(I_{n}-zA_{T}\right)}^{-1}B_{T}+D\\ \; & =zC_{T}{\left(I_{n}-zA_{T}\right)}^{-1}B_{T}+D_{T}. \end{array}$$

This is the generating function for the impulse response with the *z*-transform $F_{T}\left(1/z\right)$ which corresponds to the LDTI system

(126) $$x_{k+1}=A_{T}x_{k}+B_{T}\varpi_{k},\;\zeta_{k}=C_{T}x_{k}+D_{T}\varpi_{k}$$

with an $R^{n}$-valued state sequence $x:={\left(x_{k}\right)}_{k\in Z}$ and the state-space realization matrices

(127) $$\begin{array}{cc} A_{T}\; & :=\left(I_{n}+TA\right){\left(I_{n}-TA\right)}^{-1}=K\left(-TA\right), \end{array}$$

(128) $$\begin{array}{cc} B_{T}\; & :=T{\left(I_{n}-TA\right)}^{-1}B, \end{array}$$

(129) $$\begin{array}{cc} C_{T}\; & :=2C{\left(I_{n}-TA\right)}^{-1}, \end{array}$$

(130) $$\begin{array}{cc} D_{T}\; & :=TC{\left(I_{n}-TA\right)}^{-1}B+D, \end{array}$$

where the function *K* from (90) is evaluated [63] in (127) at the matrix −TA, with TA being Hurwitz. The system (126) can be viewed as a finite-difference scheme for numerical integration of the SDEs (7) and (11) by the trapezoidal rule with stepsize 2T. However, such interpretation assumes smallness of the time scale parameter *T* (for example, in the sense of the first relation in (89)), whereas the conformal correspondence between the LCTI and LDTI setups is valid for any T>0.

**Figure 5:**
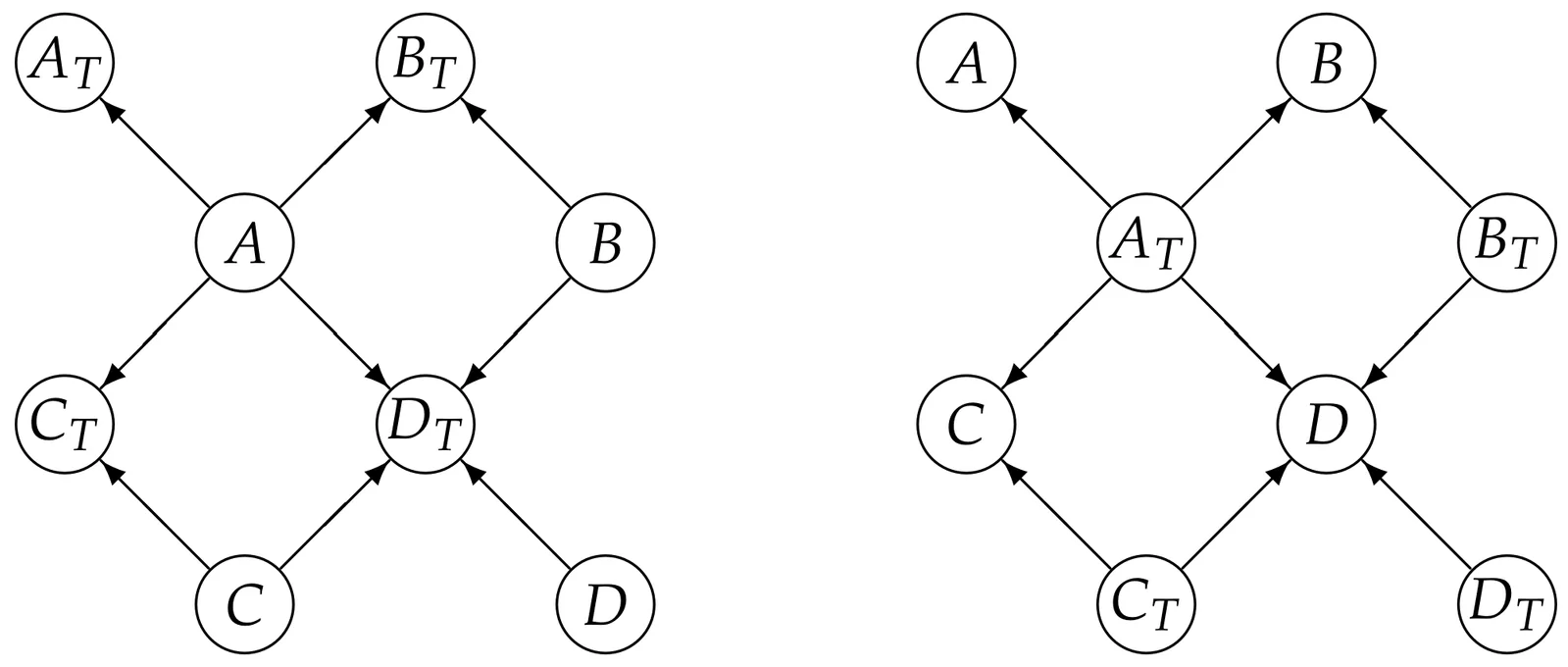
The dependencies between the matrices under the map Y in (131) described by (127)–(130) (the graph on the left) and its inverse $Y^{-1}$ in (132)–(136) (the graph on the right). Here, the arrow ◯u⟶◯v indicates dependence of *v* on *u*. For example, under the map Y, the matrix $A_{T}$ depends only on *A*, the matrix $B_{T}$ depends on *A* and *B*, while the matrix $D_{T}$ depends on all four matrices A,B,C,D.

Now, for a fixed but otherwise arbitrary T>0, the relations (127)–(130) describe a smooth bijection

(131) $$Y:\left(A,B,C,D\right)\mapsto \left(A_{T},B_{T},C_{T},D_{T}\right)$$

between the open sets of matrix quadruples such that *A* is Hurwitz and $r(A_{T})<1$. The structure of dependencies between the matrices under the map Y is shown in Figure 5 and is inherited by the inverse map

(132) $$Y^{-1}:\left(A_{T},B_{T},C_{T},D_{T}\right)\mapsto \left(A,B,C,D\right),$$

which, in view of (25), takes the form

(133) $$\begin{array}{cc} A\; & =\Omega {\left(I_{n}+A_{T}\right)}^{-1}\left(A_{T}-I_{n}\right)=-\Omega K\left(A_{T}\right), \end{array}$$

(134) $$\begin{array}{cc} B\; & =2\Omega {\left(I_{n}+A_{T}\right)}^{-1}B_{T}, \end{array}$$

(135) $$\begin{array}{cc} C\; & =C_{T}{\left(I_{n}+A_{T}\right)}^{-1}, \end{array}$$

(136) $$\begin{array}{cc} D\; & =D_{T}-C_{T}{\left(I_{n}+A_{T}\right)}^{-1}B_{T}. \end{array}$$

Relations, similar to (125) and (127)–(136), also hold for the transfer function $G_{T}$ of the discrete-time noise shaping filter in (94). As in the discrete-time case, the computation of the (T,a)-anisotropic norm (124) for the LCTI system *F* being considered is of interest only if *F* is nonround and the mean anisotropy level *a* is strictly positive (otherwise, the norm reduces trivially to the weighted $H_{2}$-norm $\frac{1}{\sqrt{m}}{∥F_{T}∥}_{2}$).

**Theorem** **4.** *Suppose the system F in (7) and (11) is stable and nonround. Then its (T,a)-anisotropic norm (124) can be computed for any given T>0 and a>0 as*

(137) $$\left||\right|F{\left||\right|}_{T,a}=\sqrt{\frac{1}{q}\left(1-\frac{m}{\mathrm{Tr}(L_{T}P_{T}L_{T}^{T}+M_{T}^{2})}\right)}.$$

*Here, $P_{T}=P_{T}^{T}\in R^{n\times n}$ is a positive semi-definite matrix which is the controllability Gramian for the pair*

(138) $$\alpha_{T}:=A_{T}+B_{T}L_{T},\;\beta_{T}:=B_{T}M_{T}$$

*and is a unique solution of the discrete algebraic Lyapunov equation (ALE)*

(139) $$P_{T}=\alpha_{T}P_{T}\alpha_{T}^{T}+\beta_{T}\beta_{T}^{T},$$

*where the matrices $A_{T},B_{T},C_{T},D_{T}$ are given by (127)–(130), and the matrices $L_{T}\in R^{m\times n}$ and $0≺M_{T}=M_{T}^{T}\in R^{m\times m}$ are associated with a unique admissible solution $R_{T}=R_{T}^{T}\in R^{n\times n}$ of the discrete algebraic Riccati equation (ARE)*

(140) $$\begin{array}{cc} R_{T}\; & =A_{T}^{T}R_{T}A_{T}+qC_{T}^{T}C_{T}+L_{T}^{T}M_{T}^{-2}L_{T}, \end{array}$$

(141) $$\begin{array}{cc} L_{T}\; & :=M_{T}^{2}\left(B_{T}^{T}R_{T}A_{T}+qD_{T}^{T}C_{T}\right), \end{array}$$

(142) $$\begin{array}{cc} M_{T}\; & :={\left(I_{m}-qD_{T}^{T}D_{T}-B_{T}^{T}R_{T}B_{T}\right)}^{-1/2}. \end{array}$$

*The admissibility of $R_{T}$ is understood in the sense that $I_{m}-qD_{T}^{T}D_{T}-B_{T}^{T}R_{T}B_{T}≻0$ is satisfied together with the stability condition*

(143) $$r(\alpha_{T})<1,$$

*and the parameter*

(144) $$0<q<\frac{1}{{∥F∥}_{\infty }^{2}}$$

*(on which the matrices $R_{T},L_{T},M_{T},P_{T}$ also depend) is a unique solution of the equation*

(145) $$-\frac{1}{2}\ln\det\left(\frac{m}{\mathrm{Tr}(L_{T}P_{T}L_{T}^{T}+M_{T}^{2})}M_{T}^{2}\right)=a.$$

*The worst-case noise spectral density is unique up to a multiplicative positive constant and is given by*

(146) $$S\left(\omega \right)={\left(I_{m}-q\Lambda \left(\omega \right)\right)}^{-1},\;\omega \in R,$$

*in terms of (64). It is delivered by an input disturbance W according to the SDE (19), where the matrices $L\in R^{m\times n}$ and $M\in R^{m\times m}$ (with M not necessarily symmetric) are computed as*

(147) $$L=L_{T}{\left(I_{n}+\alpha_{T}\right)}^{-1},\;M=M_{T}-L\beta_{T}.$$

**Figure 6:**
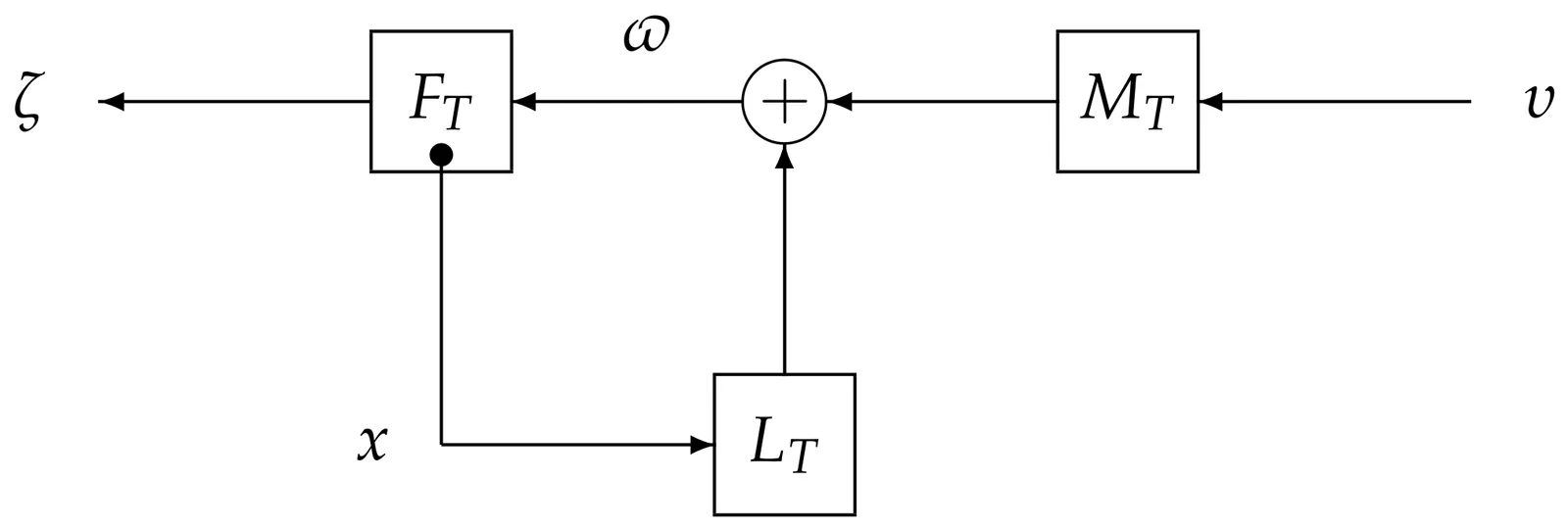
The worst-case noise shaping filter for the LDTI system $F_{T}$ with a “parasitic” feedback loop, through which the state $x_{k}$ of the system enters the predictable part $L_{T}x_{k}$ of the Doob decomposition (113), (114) and (156) of the input disturbance $\varpi_{k}$.

**Proof.**  In regard to computing the (T,a)-anisotropic norm (124) of the LCTI system *F*, the assertion of the theorem is obtained by applying the discrete-time result of [18] [Theorem 2] to the LDTI system $F_{T}$ in (126)–(130). However, for a proper adaptation of that result to the continuous-time setting, we will provide its key points on the probabilistic structure of the worst-case disturbance. To this end, we note that the stability and nonroundness of the system *F* are equivalent to the corresponding properties of its discrete-time counterpart $F_{T}$. Furthermore, both systems have equal $H_{\infty }$-norms in the appropriate Hardy spaces associated with the right half-plane and the unit disk: ${∥F∥}_{\infty }={∥F_{T}∥}_{\infty }$. Recall that the accompanying material of [Section 5] in [18] (given in more detail in [Section 8] of [21]) describes the structure

(148) $$\varpi_{k}=L_{T}x_{k}+M_{T}\upsilon_{k}$$

of the input disturbance of the discrete-time system (see Figure 6), implementing the worst-case spectral density $S_{T}$ in (96), which is unique up to a multiplicative positive constant [18] [Theorem 1] and has the form

(149) $$S_{T}\left(\varphi \right)={\left(I_{m}-q\Lambda_{T}\left(\varphi \right)\right)}^{-1},\;\varphi \in T.$$

Here, the parameter *q*, satisfying (144), is found from the saturation $\bar{A}\left(S_{T}\right)=a$ of the constraint $\bar{A}\left(S_{T}\right)⩽a$ on the mean anisotropy (108) in (123) and (124).

The representation (149) of $S_{T}$ in terms of $\Lambda_{T}$ from (95) is equivalent to the isometric property of an auxiliary LDTI system

(150) $$\Theta_{T}:=\left[\begin{array}{c} \sqrt{q}F_{T}\\ G_{T}^{-1} \end{array}\right]↔\left[\begin{array}{cc} A_{T} & B_{T}\\ \sqrt{q}C_{T} & \sqrt{q}D_{T}\\ -\rho_{T} & M_{T}^{-1} \end{array}\right],$$

which employs a matrix

(151) $$\rho_{T}:=M_{T}^{-1}L_{T}$$

and has the input ϖ and the output

$$\left[\begin{array}{c} \sqrt{q}\zeta \\ \upsilon \end{array}\right].$$

The right-hand side of (150) describes the state-space realization matrices of the system $\Theta_{T}$, with its bottom block row corresponding to the equation

$$\upsilon_{k}=-\rho_{T}x_{k}+M_{T}^{-1}\varpi_{k}$$

obtained from (148) due to $\det M_{T}\neq 0$. The condition that the system $\Theta_{T}$ is isometric is understood in the sense that if ϖ were an arbitrary square summable sequence in $R^{m}$ (instead of being a stationary Gaussian sequence whose sample paths are square summable only in the trivial case ϖ=0) and hence, so also were υ, ζ in (102) and (104), then their $\ell_{2}$-norms would be related by ${∥\varpi ∥}_{2}^{2}={q∥\zeta ∥}_{2}^{2}+{∥\upsilon ∥}_{2}^{2}$. However, in the case of the worst-case stationary Gaussian sequence ϖ being considered, the isometric property of the system (150) manifests itself in terms of the variances as

$$E({|\varpi_{k}|}^{2})=qE({\left|\zeta_{k}\right|}^{2})+m,$$

where use is also made of the relation $E(|\upsilon_{k}{|}^{2})=\mathrm{Tr}I_{m}=m$ which follows from (103). With the matrix $R_{T}$ in (140) being the observability Gramian of the system (150), the other two equations in the ARE (140)–(142) describe a sufficient state-space condition for the system $\Theta_{T}$ to be isometric [18]. This condition is also necessary if the pair $\left(A_{T},B_{T}\right)$ is controllable [64]. Similarly to its inverse $G_{T}^{-1}$ in (150), the shaping filter

(152) $$G_{T}↔\left[\begin{array}{cc} \alpha_{T} & \beta_{T}\\ L_{T} & M_{T} \end{array}\right],$$

governed by the first of the Equations (126) in combination with (148), shares the common state *x* with the system $F_{T}$:

(153) $$x_{k+1}=\alpha_{T}x_{k}+\beta_{T}\upsilon_{k},\;\varpi_{k}=L_{T}x_{k}+M_{T}\upsilon_{k},$$

where the matrices $\alpha_{T}$, $\beta_{T}$ are given by (138) and, as before, the input of the filter is the Gaussian white-noise sequence υ in $R^{m}$ with zero mean and identity covariance matrix. The stability of the filter (153) is secured by the condition (143) and, in combination with (103), leads to the Equation (139) for the covariance matrix

(154) $$P_{T}:=\mathrm{cov}\left(x_{k}\right)$$

of the state of the LDTI system $F_{T}$ under the worst-case input disturbance (148) being considered. Accordingly, the covariance matrix

(155) $$\mathrm{cov}\left(\varpi_{k}\right)=L_{T}P_{T}L_{T}^{T}+M_{T}^{2}$$

of the output of the discrete-time noise shaping filter (152) gives rise to its variance in the common denominator of (137) and (145):

$$E(|\varpi_{k}{|}^{2})=\mathrm{Tr}\left(L_{T}P_{T}L_{T}^{T}+M_{T}^{2}\right)$$

in view of the symmetry $M_{T}=M_{T}^{T}$. Due to the stability of the shaping filter $G_{T}$ and the system $F_{T}$ itself (recall that (143) is fulfilled along with $r(A_{T})<1$), and also since $\det M_{T}\neq 0$, the sequence ϖ generates the same filtration F as υ, with (148) providing its Doob decomposition (114) into the F-predictable and innovation components $\hat{\varpi }$, $\tilde{\varpi }$ in (113) as

(156) $${\hat{\varpi }}_{k}=L_{T}x_{k},\;{\tilde{\varpi }}_{k}=M_{T}\upsilon_{k}.$$

Hence, with the matrix $M_{T}$ being symmetric, the one-step prediction error covariance matrix (112) takes the form

(157) $$\Gamma =\mathrm{cov}\left(M_{T}\upsilon_{k}|F_{k-1}\right)=M_{T}\mathrm{cov}\left(\upsilon_{k}\right)M_{T}^{T}=M_{T}^{2},$$

thus leading to the numerator $M_{T}^{2}$ in (145). The structure of the equivalent continuous-time input disturbance *W* is obtained by applying the inverse map $Y^{-1}$ from (132) (in accordance with (133)–(136)) to the worst-case shaping filter (152), which leads to (19) with the matrices (147). Therefore, in view of (11), the worst-case continuous-time noise shaping filter *G* is governed by

(158) dX=αXdt+βdV,dW=LXdt+MdV,

so that

(159) $$G↔\left[\begin{array}{cc} \alpha & \beta \\ L & M \end{array}\right],$$

where

(160) α:=A+BL,β:=BM

in accordance with (20). In view of the conformal correspondence (95) and (96), the worst-case noise spectral density is obtained from (149) and takes the form of (146). Due to the properties of the Cayley transform (90) and the map $Y^{-1}$ (see also Figure 5), the dynamics matrix α in (158)–(160) is related to its discrete-time counterpart $\alpha_{T}$ in (138), (152) and (153) by

$$\alpha =\Omega {\left(I_{n}+\alpha_{T}\right)}^{-1}\left(\alpha_{T}-I_{n}\right)=-\Omega K\left(\alpha_{T}\right)$$

and, in view of the condition (143), is Hurwitz. The latter follows from (91) and the transformation of the spectrum for a function of a matrix [63]. □

Note that, by the same conformal correspondence argument as in the proof of Theorem 4 (using the bijection between the unit circle and the imaginary axis), the LCTI system

(161) $$\Theta :=\left[\begin{array}{c} \sqrt{q}F\\ G^{-1} \end{array}\right]↔\left[\begin{array}{cc} A & B\\ \sqrt{q}C & \sqrt{q}D\\ -\rho & M^{-1} \end{array}\right],$$

which involves an auxiliary matrix

(162) $$\rho :=M^{-1}L$$

and is associated with the worst-case disturbance *W* described in the theorem, inherits the property of being inner from its discrete-time counterpart $\Theta_{T}$ in (150), so that its transfer function satisfies

$$\Theta {\left(i\omega \right)}^{*}\Theta \left(i\omega \right)=I_{m},\;\omega \in R.$$

Moreover, the structure (146) of the worst-case noise spectral density *S* is equivalent to the innerness of Θ. In particular, by continuity, the feedthrough matrix in (161) describes an isometric embedding from $R^{m}$ into $R^{p+m}$:

(163) $$\begin{array}{cc} \lim_{\omega \to \infty }\left(\Theta {\left(i\omega \right)}^{*}\Theta \left(i\omega \right)\right)\; & =\left[\begin{array}{cc} \sqrt{q}D^{T} & M^{-T} \end{array}\right]\left[\begin{array}{c} \sqrt{q}D\\ M^{-1} \end{array}\right]\\ \; & =qD^{T}D+{\left(MM^{T}\right)}^{-1}=I_{m}, \end{array}$$

where $M^{-T}:={\left(M^{-1}\right)}^{T}$. From (163), or from a combination of (146) with (64), (37), (40) and (159), it follows that

(164) $$\Delta :=MM^{T}=\lim_{\omega \to \infty }S\left(\omega \right)={\left(I_{m}-qD^{T}D\right)}^{-1}.$$

The matrix on the right-hand side of (164) is well-defined and positive definite. Indeed, $\lambda_{\max}\left(D^{T}D\right)⩽{∥F∥}_{\infty }^{2}$ and hence, (144) implies that $q<\frac{1}{{∥F∥}_{\infty }^{2}}⩽\frac{1}{\lambda_{\max}\left(D^{T}D\right)}$, whereby $qD^{T}D≺I_{m}$ and Δ≻0.

As discussed above, similarly to the discrete-time representation in Figure 6, the worst-case disturbance *W* at the input of the LCTI system *F* in (7) and (11) is formed in (19) by the feedback loop (see Figure 2), through which the system state *X* enters the drift LX of the disturbance. In line with this, we note that the stability condition (that the matrix α in (158)–(160) has to be Hurwitz) and the mean anisotropy constraint in (123) forbid the hypothetical “noise player” to use an arbitrarily large matrix *L* for destabilizing the system and force the opponent to mix the predictable component $L\int_{0}^{t}X\left(\tau \right)d\tau$ of the disturbance *W* with the innovation process MV.

Accordingly, the matrix Δ in (164), which is the diffusion matrix of the worst-case noise *W* governed by (158), plays a role similar to that of Γ in (157). Indeed, both matrices quantify the second moments of the innovation components MdV and $M_{T}\upsilon_{k}$ of the input disturbances in the continuous and discrete-time representations (158) and (153), respectively.

In the worst-case noise scenario at the mean anisotropy level a⩾0, the latter admits the decomposition

(165) $$a=a_{\mathrm{spat}}+a_{\mathrm{temp}}$$

into the spatial and temporal components which are found from (145) according to (121) and (122) as

(166) $$\begin{array}{cc} a_{\mathrm{spat}}\; & =-\frac{1}{2}\ln\det\left(\frac{m}{\mathrm{Tr}(L_{T}P_{T}L_{T}^{T}+M_{T}^{2})}\left(L_{T}P_{T}L_{T}^{T}+M_{T}^{2}\right)\right)\\ \; & =-\frac{1}{2}\ln\det\left(\frac{m}{\langle M_{T}^{2},I_{m}+\Xi_{T}\Xi_{T}^{T}\rangle }\left(I_{m}+\Xi_{T}\Xi_{T}^{T}\right)M_{T}^{2}\right), \end{array}$$

(167) $$\begin{array}{cc} a_{\mathrm{temp}}\; & =\frac{1}{2}\ln\det\left(M_{T}^{-2}\left(L_{T}P_{T}L_{T}^{T}+M_{T}^{2}\right)\right)\\ \; & =\frac{1}{2}\ln\det\left(I_{m}+\Xi_{T}\Xi_{T}^{T}\right), \end{array}$$

where

(168) $$\Xi_{T}:=\rho_{T}\sqrt{P_{T}}.$$

Here, use has been made of the matrix $\rho_{T}$ from (151) along with the fact that the matrix

$$L_{T}P_{T}L_{T}^{T}+M_{T}^{2}=M_{T}\left(I_{m}+\Xi_{T}\Xi_{T}^{T}\right)M_{T}^{2}M_{T}^{-1}$$

in (155) is related by a similarity transformation and is therefore isospectral to the matrix

$$\left(I_{m}+\Xi_{T}\Xi_{T}^{T}\right)M_{T}^{2}.$$

Note that the temporal part $a_{\mathrm{temp}}$ in (167) is completely specified by the singular values of the matrix $\Xi_{T}$ from (168).

While the mean anisotropy functional (108) and its spatio-temporal decomposition (120)–(122) have an entropy-theoretic interpretation in terms of the auxiliary Gaussian sequence (100) discussed in Section 8, and $a_{\mathrm{spat}}$ in (166) admits two-sided bounds [21] [p. 31] in terms of the condition number of the matrix (155), it is relevant to link the temporal component $a_{\mathrm{temp}}$ in (167) with relative entropy rates for the worst-case noise *W* in its native continuous time.

## 10. Relative Entropy Rates for Worst-Case Disturbance

Consider the system *F* subject to the worst-case noise *W* from Theorem 4 (at a given mean anisotropy level a>0) governed by the SDEs (158) with zero initial conditions W(0)=0 and V(0)=0. For any t⩾0, the restriction ${W|}_{\left[0,t\right]}$ of *W* to the time interval [0,t] is a random element with values in the Banach space $C_{0}\left(\left[0,t\right],R^{m}\right)$ of continuous functions $w:\left[0,t\right]\to R^{m}$ satisfying w(0)=0 and equipped with the uniform norm. By Girsanov’s theorem [45], the probability distribution of ${W|}_{\left[0,t\right]}$ on Borel subsets of $C_{0}\left(\left[0,t\right],R^{m}\right)$ is absolutely continuous with respect to that of the Wiener process MV with the diffusion matrix Δ≻0 in (164), and its Radon-Nikodym derivative, evaluated at ${W|}_{\left[0,t\right]}$, is found from

(169) $$\begin{array}{cc} \ln R(t)\; & =\int_{0}^{t}X{\left(\tau \right)}^{T}L^{T}\Delta^{-1}dW\left(\tau \right)-\frac{1}{2}\int_{0}^{t}{∥LX\left(\tau \right)∥}_{\Delta^{-1}}^{2}d\tau \\ \; & =\int_{0}^{t}X{\left(\tau \right)}^{T}\rho^{T}dV\left(\tau \right)+\frac{1}{2}\int_{0}^{t}{\left|\rho X\left(\tau \right)\right|}^{2}d\tau , \end{array}$$

where use is also made of the matrix ρ from (162). The validity of the representation (169) is guaranteed whenever an appropriately modified version of Novikov’s condition [44] is satisfied:

(170) $$E_{*}e^{\frac{1}{2}\int_{0}^{t}{\left|\rho X\left(\tau \right)\right|}^{2}d\tau }<+\infty ,$$

where $E_{*}\left(\cdot \right)$ is the expectation over a reference probability measure $P_{*}$ which considers $M^{-1}W$ a standard Wiener process in $R^{m}$. The fulfillment of (170) for any t>0 secures the martingale property (in the sense of $P_{*}$) on any bounded time interval for the random process *R* in (169) with respect to the natural filtration of *W*. Returning to the actual probability measure P with the expectation E(·), according to which *W* is governed by (158) where *V* is a standard Wiener process, we note that (169) allows the corresponding Renyi’s relative entropy [55] of order 1+ϵ to be computed for any ϵ>0 small enough (in the sense specified below) as

(171) $$\begin{array}{cc} R_{\epsilon }\left(t\right)\; & :=\frac{1}{\epsilon }\ln E\left(R{\left(t\right)}^{\epsilon }\right)=\frac{1}{\epsilon }\ln Ee^{\epsilon \ln R(t)}\\ \; & =\frac{1}{\epsilon }\ln Ee^{\epsilon \int_{0}^{t}X{\left(\tau \right)}^{T}\rho^{T}dV\left(\tau \right)+\frac{\epsilon }{2}\int_{0}^{t}{\left|\rho X\left(\tau \right)\right|}^{2}d\tau }\\ \; & =\frac{1}{\epsilon }\ln E\left(\mu_{\epsilon }\left(t\right)e^{\frac{1}{2}\epsilon \left(1+\epsilon \right)\int_{0}^{t}{\left|\rho X\left(\tau \right)\right|}^{2}d\tau }\right)\\ \; & =\frac{1}{\epsilon }\ln Ee^{\frac{1}{2}\epsilon \left(1+\epsilon \right)\int_{0}^{t}{\left|\rho X\left(\tau \right)\right|}^{2}d\tau }. \end{array}$$

Here,

(172) $$\mu_{\epsilon }\left(t\right):=e^{\epsilon \int_{0}^{t}X{\left(\tau \right)}^{T}\rho^{T}dV\left(\tau \right)-\frac{1}{2}\epsilon^{2}\int_{0}^{t}{\left|\rho X\left(\tau \right)\right|}^{2}d\tau },$$

and use is made of its martingale property on any bounded time interval under the sufficient condition

(173) $$Ee^{\frac{1}{2}\epsilon^{2}\int_{0}^{t}{\left|\rho X\left(\tau \right)\right|}^{2}d\tau }<+\infty ,\;t>0.$$

The KL relative entropy of the probability distribution of ${W|}_{\left[0,t\right]}$ with respect to that of ${MV|}_{\left[0,t\right]}$ is recovered from (171) and (169) as

(174) $$\begin{array}{cc} R_{0}\left(t\right)\; & :=\lim_{\epsilon \to 0+}R_{\epsilon }\left(t\right)=E\ln R\left(t\right)\\ \; & =\frac{1}{2}\int_{0}^{t}{E(|\rho X\left(\tau \right)|}^{2})d\tau ⩽R_{\epsilon }\left(t\right), \end{array}$$

where the inequality (provided here for completeness of exposition) follows from Jensen’s inequality.

Note that the relative entropy (171) (and its limiting case (174)) takes the same value for the probability law of the filtered noise $W_{T}{|}_{\left[0,t\right]}$ with respect to that of $MV_{T}{|}_{\left[0,t\right]}$ because $W_{T}$ is obtained from *W* by the same bijective causal transformation as $MV_{T}$ from MV as specified by (22) and (23), and this transformation preserves the natural filtration of the processes.

The theorem below on the relative entropy rates for the worst-case noise *W* employs the transfer function

(175) $$N\left(s\right):={\left(sI_{n}-\alpha \right)}^{-1}\beta ,\;s\in C$$

of an auxiliary LCTI system which maps *V* to the state process *X* governed by the first SDE in (158) with the Hurwitz matrix α. In this worst-case noise scenario, the invariant covariance matrix of the process *X*, given by

(176) $$P:=\lim_{t\to +\infty }P\left(t\right)=\int_{R_{+}}e^{t\alpha }\beta \beta^{T}e^{t\alpha^{T}}dt=\frac{1}{2\pi }\int_{R}N\left(i\omega \right)N{\left(i\omega \right)}^{*}d\omega ,$$

coincides with the controllability Gramian of the pair (α,β) from (160) and satisfies a continuous ALE

(177) $$\alpha P+P\alpha^{T}+\beta \beta^{T}=0.$$

Here, the covariance matrix

(178) $$P\left(t\right):=\mathrm{cov}\left(X\left(t\right)\right)=\int_{0}^{t}e^{\tau \alpha }\beta \beta^{T}e^{\tau \alpha^{T}}d\tau =P-e^{t\alpha }Pe^{t\alpha^{T}},\;t⩾0,$$

converges to its limit *P* in (176) monotonically, so that 0≼P(u)≼P(s)≼P for any s⩾u⩾0.

**Theorem** **5.** *Suppose the system F in (7) and (11) is subject to the worst-case noise W (at a given mean anisotropy level a>0) governed by the SDEs (158) as described in Theorem 4 and its proof. Then for any ϵ satisfying*

(179) $$0<\epsilon <\frac{1}{2}\left(\sqrt{1+{4∥\rho N∥}_{\infty }^{-2}}-1\right)$$

*in terms of the matrix ρ from (162) and the $H_{\infty }$-norm of the transfer function (175), the Renyi and KL relative entropies (171) and (174) have the following rates:*

(180) $$\begin{array}{cc} \lim_{t\to +\infty }\frac{R_{\epsilon }\left(t\right)}{t}\; & =-\frac{1}{4\pi \epsilon }\int_{R}\ln\det\left(I_{m}-\epsilon \left(1+\epsilon \right)\rho N\left(i\omega \right)N{\left(i\omega \right)}^{*}\rho^{T}\right)d\omega \\ \; & ⩾\lim_{t\to +\infty }\frac{R_{0}\left(t\right)}{t}=\frac{1}{2}{∥\Xi ∥}_{F}^{2}, \end{array}$$

*where*

(181) $$\Xi :=\rho \sqrt{P},$$

*and P is the matrix from (176) and (177).*

**Proof.**  The zero-initialized Gaussian state process *X* in (158) has zero mean and a continuous covariance function

(182) $$\varkappa \left(s,u\right):=\mathrm{cov}\left(X\left(s\right),X\left(u\right)\right)=\left\{\begin{array}{ccc} e^{(s-u)\alpha }P\left(u\right) & \mathrm{for} & s⩾u⩾0\\ P\left(s\right)e^{\left(u-s\right)\alpha^{T}} & \mathrm{for} & u>s⩾0 \end{array}\right.,$$

where use is made of the covariance matrix from (178). By the convergence (176), the resulting limit of (182) takes the form

(183) $$\begin{array}{cc} \varkappa_{\infty }\left(\tau \right)\; & :=\lim_{u\to +\infty }\varkappa \left(u+\tau ,u\right)=\left\{\begin{array}{ccc} e^{\tau \alpha }P & \mathrm{if} & \tau ⩾0\\ Pe^{-\tau \alpha^{T}} & \mathrm{if} & \tau <0 \end{array}\right.\\ \; & =\frac{1}{2\pi }\int_{R}e^{i\omega \tau }N\left(i\omega \right)N{\left(i\omega \right)}^{*}d\omega ,\;\tau \in R, \end{array}$$

which depends only on the time difference and is the covariance function of the process *X* in its stationary regime, where *N* is the transfer function from (175). For a fixed but otherwise arbitrary time horizon t>0, the function (182) gives rise to a compact positive semi-definite self-adjoint integral operator $H_{t}$ on the Hilbert space $L^{2}\left(\left[0,t\right],R^{m}\right)$, whose $R^{m\times m}$-valued kernel function on the square ${\left[0,t\right]}^{2}$ is the covariance function $\rho \varkappa \rho^{T}$ of the process ρX, so that, similarly to (3),

(184) $$\begin{array}{cc} E\left(\int_{0}^{t}\varphi {\left(s\right)}^{T}\rho X\left(s\right)ds\int_{0}^{t}\psi {\left(u\right)}^{T}\rho X\left(u\right)du\right)\; & =\int_{0}^{t}\varphi {\left(s\right)}^{T}H_{t}\left(\psi \right)\left(s\right)ds,\\ H_{t}\left(\psi \right)\left(s\right)\; & :=\rho \int_{0}^{t}\varkappa \left(s,u\right)\rho^{T}\psi \left(u\right)du,\;0⩽s⩽t, \end{array}$$

for any $\varphi ,\psi \in L^{2}\left(\left[0,t\right],R^{m}\right)$. The operator $H_{t}$ is majorized by a compact positive semi-definite self-adjoint Toeplitz integral operator $K_{t}$ on $L^{2}\left(\left[0,t\right],R^{m}\right)$ with the kernel function $\rho \varkappa_{\infty }\rho^{T}$ associated with (183):

(185) $$H_{t}≼K_{t}.$$

The $L^{2}$-induced operator norms of $H_{t}$ and $K_{t}$ coincide with their largest eigenvalues and are nondecreasing functions of time *t* satisfying

(186) $$\begin{array}{cc} \lambda_{\max}\left(H_{t}\right)\; & ⩽\lambda_{\max}\left(K_{t}\right)⩽\lim_{\tau \to +\infty }\lambda_{\max}\left(K_{\tau }\right)\\ \; & =\underset{\omega \in R}{\sup}\lambda_{\max}\left(\rho N\left(i\omega \right)N{\left(i\omega \right)}^{*}\rho^{T}\right)={∥\rho N∥}_{\infty }^{2}. \end{array}$$

Here, the first inequality follows from (185), while the first equality follows from the fact that the function $R∋\omega \mapsto \rho N\left(i\omega \right)N{\left(i\omega \right)}^{*}\rho^{T}\in H_{m}^{+}$ is the Fourier transform of the kernel function $\rho \varkappa_{\infty }\rho^{T}$ of the operator $K_{t}$ in view of (183) and from the asymptotic properties of spectra of Toeplitz operators [62]. Hence, for any ϵ subject to (179), where the second inequality originates from the condition

(187) $$\epsilon \left(1+\epsilon \right)<\frac{1}{{∥\rho N∥}_{\infty }^{2}},$$

the operator $\epsilon \left(1+\epsilon \right)H_{t}$ is a contraction, and so also is $\epsilon^{2}H_{t}$. In combination with the fact that *X* is a zero-mean Gaussian process, the relations (186) and (187) secure the fulfillment of the sufficient condition (173) for the martingale property of (172), which is used in (171), and make the right-hand side of (171) well-defined. Application of the results of [65] (see also the references therein) and the Fredholm formula [66] [Theorem 3.10 on p. 36] to the right-hand side of (171) yields

(188) $$\begin{array}{cc} R_{\epsilon }\left(t\right)\; & =-\frac{1}{2\epsilon }\mathrm{Tr}\ln\left(I-\epsilon \left(1+\epsilon \right)H_{t}\right)\\ \; & =\frac{1}{2}\sum_{k=1}^{+\infty }\frac{1}{k}\epsilon^{k-1}{\left(1+\epsilon \right)}^{k}\mathrm{Tr}\left(H_{t}^{k}\right),\;t>0, \end{array}$$

where I is the identity operator. Since the function $Q\mapsto -\mathrm{Tr}\ln\left(I-Q\right)\in R_{+}$ is monotonic with respect to a self-adjoint operator 0≼Q≺I of finite trace, it follows from (185)–(188) that

$$R_{\epsilon }\left(t\right)⩽-\frac{1}{2\epsilon }\mathrm{Tr}\ln\left(I-\epsilon \left(1+\epsilon \right)K_{t}\right).$$

Hence,

(189) $$\begin{array}{cc} \underset{t\to +\infty }{\mathrm{lim sup}}\frac{R_{\epsilon }\left(t\right)}{t}\; & ⩽-\frac{1}{2\epsilon }\lim_{t\to +\infty }\left(\frac{1}{t}\mathrm{Tr}\ln\left(I-\epsilon \left(1+\epsilon \right)K_{t}\right)\right)\\ \; & =-\frac{1}{4\pi \epsilon }\int_{R}\ln\det\left(I_{m}-\epsilon \left(1+\epsilon \right)\rho N\left(i\omega \right)N{\left(i\omega \right)}^{*}\rho^{T}\right)d\omega , \end{array}$$

where the last equality follows from the Szegö limit theorem for Toeplitz operators [62]. On the other hand, (171) implies that

(190) $$\begin{array}{cc} R_{\epsilon }\left(t\right)\; & ⩾\frac{1}{\epsilon }\ln Ee^{\frac{1}{2}\epsilon \left(1+\epsilon \right)\int_{\tau }^{t}{\left|\rho X\left(s\right)\right|}^{2}ds}\\ \; & =-\frac{1}{2\epsilon }\mathrm{Tr}\ln\left(I-\epsilon \left(1+\epsilon \right)H_{\tau ,t}\right),\;0⩽\tau <t, \end{array}$$

where $H_{\tau ,t}$ is the covariance operator of the process ρX on the time interval [τ,t]. More precisely, $H_{\tau ,t}$ is an integral operator on the space

$$L^{2}\left(\left[\tau ,t\right],R^{m}\right)=T_{\tau }\left(L^{2}\left(\left[0,t-\tau \right],R^{m}\right)\right)$$

with the kernel function $\rho \varkappa \rho^{T}$ on ${\left[\tau ,t\right]}^{2}$, where $T_{\tau }$ is the unitary operator of translation by τ acting on a function φ as $T_{\tau }\left(\varphi \right)\left(s\right)=\varphi \left(s-\tau \right)$, so that its adjoint is $T_{\tau }^{†}=T_{-\tau }$. In particular, $H_{0,t}=H_{t}$ as defined in (184). Due to the Hurwitz property of the matrix α in (160) leading to the monotonic convergence in (176), for any given δ∈(0,1], there exists τ⩾0 such that the function (178) satisfies

(191) P(t)≽P(τ)≽(1−δ)P,t⩾τ.

This property can be verified (in the general case, when the pair (α,β) is not necessarily controllable and *P* in (176) can be singular) by decomposing the state space into the orthogonal sum $R^{n}=\ker P⊕\mathrm{im}P$ and noting that

$$x^{T}e^{\tau \alpha }Pe^{\tau \alpha^{T}}x={\left|\sqrt{P}e^{\tau \alpha^{T}}x\right|}^{2}=0,\;x\in \ker P$$

for any τ⩾0, while the matrix *P* is positive definite on the reachable subspace imP, whereby $\sqrt{P}x\neq 0$ for any x∈imP∖{0}. Therefore, (191) is equivalent to

$$\max_{x\in \mathrm{im}P:\;|\sqrt{P}x|=1}\left|\sqrt{P}e^{\tau \alpha^{T}}x\right|⩽\sqrt{\delta }$$

and can indeed be satisfied for any given δ>0 by choosing τ large enough due to α being Hurwitz. By the dependence of the covariance function (182) on P in (178), the fulfillment of the condition (191) implies that

(192) $$H_{\tau ,t}≽\left(1-\delta \right)T_{\tau }K_{t-\tau }T_{-\tau },\;t>\tau ,$$

where use is also made of the translation invariance of the kernel function $\rho \varkappa_{\infty }\rho^{T}$ of the Toeplitz operator $K_{t}$. By combining (190) with (192) and using the monotonicity argument as before along with the unitary equivalence (and thus, isospectrality) of the operators $T_{\tau }K_{t-\tau }T_{-\tau }$ and $K_{t-\tau }$, it follows that

$$\begin{array}{cc} R_{\epsilon }\left(t\right)\; & ⩾-\frac{1}{2\epsilon }\mathrm{Tr}\ln\left(I-\epsilon \left(1+\epsilon \right)\left(1-\delta \right)T_{\tau }K_{t-\tau }T_{-\tau }\right)\\ \; & =-\frac{1}{2\epsilon }\mathrm{Tr}\ln\left(I-\epsilon \left(1+\epsilon \right)\left(1-\delta \right)K_{t-\tau }\right),\;t>\tau , \end{array}$$

and hence,

(193) $$\begin{array}{cc} \underset{t\to +\infty }{\mathrm{lim inf}}\frac{R_{\epsilon }\left(t\right)}{t}\; & ⩾-\frac{1}{2\epsilon }\lim_{t\to +\infty }\left(\frac{1}{t}\mathrm{Tr}\ln\left(I-\epsilon \left(1+\epsilon \right)\left(1-\delta \right)K_{t-\tau }\right)\right)\\ \; & =-\frac{1}{4\pi \epsilon }\int_{R}\ln\det\left(I_{m}-\epsilon \left(1+\epsilon \right)\left(1-\delta \right)\rho N\left(i\omega \right)N{\left(i\omega \right)}^{*}\rho^{T}\right)d\omega . \end{array}$$

Since δ∈(0,1] is arbitrary, then by letting δ→0+, a comparison of (193) with (189) establishes the first equality in (180). The inequality and the second equality in (180) are obtained from (174) and (176) by using (183) at τ=0 along with (181). □

In view of the last equality in (171) and the fact that the function $R∋\omega \mapsto \rho N\left(i\omega \right)N{\left(i\omega \right)}^{*}\rho^{T}\in H_{m}^{+}$ is the spectral density of the Gaussian process ρX in the stationary regime of the first SDE from (158), the first equality in (180) is a particular case of the quadratic-exponential functional rates for stationary Gaussian processes in infinite-horizon risk-sensitive control [67,68,69].

The relative entropy rates (180) have the physical dimension of the inverse of time. The reciprocal quantity

(194) $$\tau_{*}:=\frac{2}{{∥\Xi ∥}_{F}^{2}},$$

which involves the matrix (181), can be interpreted as characteristic time for the deviation of the worst-case noise *W* from the Wiener process MV to manifest itself in terms of the relative entropies (171) and (174) in the stationary regime of (158) when they take the form

$$R_{\epsilon }\left(t\right)⩾R_{0}\left(t\right)=\frac{t}{\tau_{*}},\;t⩾0.$$

Note that such deviation can be detected in the long run (at time horizons $t\gg \tau_{*}$) by statistical hypothesis testing [70,71], although detailed analysis of this issue is beyond the scope of the present paper. Also, a related quantity

(195) $$\frac{m}{{∥\Xi ∥}_{F}^{2}}=\frac{m}{2}\tau_{*}$$

describes qualitatively the local time scale at which the drift and diffusion terms in the modified form

$$M^{-1}dW=\rho Xdt+dV$$

of the second SDE from (158) make comparable contributions to the increment of the process $M^{-1}W$. This is understood at the level of the second-order moments (in the stationary regime) including the relation

(196) $$E(|V\left(s\right)-V\left(t\right){|}^{2})=m|s-t|,\;s,t\in R,$$

for the standard Wiener process *V* in $R^{m}$ from (21). More precisely, the drift-diffusion balance time (195) is obtained by formally equating mτ from (196) with $E({\left|\rho X\right|}^{2})\tau^{2}={∥\Xi ∥}_{F}^{2}\tau^{2}$ for the time difference τ:=|s−t|>0.

The structure of the matrix Ξ in (181) appearing in the KL relative entropy rate (180) is identical to that of the matrix $\Xi_{T}$ in (168) which participates in the temporal component (167) of the mean anisotropy level, with the matrices ρ, *P* in (162) and (177) and $\rho_{T}$, $P_{T}$ in (151) and (139) being counterparts of each other in continuous and discrete time, respectively. This resemblance is not coincidental and is substantiated in the proof of the following theorem.

**Theorem** **6.** *As in Theorem 5, suppose the system F is subject to the worst-case noise W governed by the SDEs (158) as specified in Theorem 4 and its proof. Then the temporal component (167) of the mean anisotropy level a>0 is linked to the KL relative entropy rate in (180) through the time scale (194) by*

(197) $$a_{\mathrm{temp}}⩽2\sigma \frac{T}{\tau_{*}},$$

*where*

(198) $$\sigma :=\lambda_{\max}\left(\Gamma^{-1}\Delta \right)$$

*is the largest eigenvalue for the linear matrix pencil Δ−λΓ associated with the matrices* Γ*,* Δ *from (157) and (164).*

**Proof.**  The inequality (197) can be established as a corollary from the intermediate inequalities

(199) $$a_{\mathrm{temp}}⩽\frac{1}{2}∥\Xi_{T}{∥}_{F}^{2}\;⩽\sigma T{∥\Xi ∥}_{F}^{2},$$

where the right-hand side coincides with that of (197) in view of (194). The first inequality in (199) is obtained by combining (167) with the fact that the function lndet(·) is concave on the set of positive definite matrices (as mentioned before) and vanishes at the identity matrix, where the Frechet derivative of this function satisfies $\partial_{Q}{\ln\det Q|}_{Q=I_{m}}=I_{m}$, so that

$$\ln\det\left(I_{m}+\Xi_{T}\Xi_{T}^{T}\right)⩽\langle I_{m},\Xi_{T}\Xi_{T}^{T}\rangle ={∥\Xi_{T}∥}_{F}^{2}.$$

We will now prove the second inequality in (199). The discrete-time state-space realization (153) gives rise to the transfer function for the map υ↦x:

(200) $$N_{T}\left(z\right):=z{\left(I_{n}-z\alpha_{T}\right)}^{-1}\beta_{T},\;z\in C,$$

with the matrices $\alpha_{T}$, $\beta_{T}$ from (138). In view of (143), the invariant covariance matrix $P_{T}$ of the stationary Gaussian sequence *x* in (154), which satisfies the discrete ALE (139), is expressed in terms of (200) as

(201) $$P_{T}=\frac{1}{2\pi }\int_{T}N_{T}\left(e^{i\varphi }\right)N_{T}{\left(e^{i\varphi }\right)}^{*}d\varphi .$$

The matrix $P_{T}$ should not be confused with the invariant covariance matrix of the filtered state process $X_{T}$ in (51), which, in the worst-case noise scenario, is governed by the ODE

(202) $${\dot{X}}_{T}=AX_{T}+B\left(LX_{T}+MV_{T}\right)=\alpha X_{T}+\beta V_{T},$$

where the matrices (160) are used, and $V_{T}$ is the OU process as before. Indeed, by combining the spectral density $\Sigma I_{m}$ of $V_{T}$ from (61) with (175), (202) and the initial condition for $X_{T}$ from (54), it follows that

(203) $$\begin{array}{cc} J_{T}\; & :=\lim_{t\to +\infty }\mathrm{cov}\left(X_{T}\left(t\right)\right)\\ \; & =\frac{1}{2\pi }\int_{R}\Sigma \left(\omega \right)N\left(i\omega \right)N{\left(i\omega \right)}^{*}d\omega \\ \; & =\frac{1}{2\pi }\int_{T}N\left(i\Omega \tan\frac{\varphi }{2}\right)N{\left(i\Omega \tan\frac{\varphi }{2}\right)}^{*}d\varphi \\ \; & =\frac{1}{2\pi }\int_{T}N\left(-i\Omega \tan\frac{\varphi }{2}\right)N{\left(-i\Omega \tan\frac{\varphi }{2}\right)}^{*}d\varphi \\ \; & =\frac{1}{2\pi }\int_{T}N\left(\Omega K\left(e^{i\varphi }\right)\right)N{\left(\Omega K\left(e^{i\varphi }\right)\right)}^{*}d\varphi \\ \; & =\frac{1}{2\pi i}\oint_{|z|=1}N\left(\Omega K\left(z\right)\right)N{\left(\Omega K\left(z\right)\right)}^{*}\frac{dz}{z}, \end{array}$$

with the integration variable change according to (84) and (92). The last integrand in (203) can be computed similarly to (125) by using

(204) $$\begin{array}{cc} N(\Omega K(z))\; & ={\left(\Omega \frac{1-z}{1+z}I_{n}-\alpha \right)}^{-1}\beta \\ \; & =\left(1+z\right){\left(I_{n}-z\alpha_{T}\right)}^{-1}\beta_{T}\\ \; & =2z{\left(I_{n}-T\alpha \right)}^{-1}{\left(I_{n}-z\alpha_{T}\right)}^{-1}\beta_{T}+T{\left(I_{n}-T\alpha \right)}^{-1}\beta \\ \; & ={\left(I_{n}-T\alpha \right)}^{-1}\left(2N_{T}\left(z\right)+T\beta \right), \end{array}$$

where the last equality involves the transfer function $N_{T}$ from (200). Substitution of (204) into (203) yields

(205) $$\begin{array}{cc} J_{T}\; & =\frac{1}{2\pi i}{\left(I_{n}-T\alpha \right)}^{-1}\oint_{|z|=1}\left(2N_{T}\left(z\right)+T\beta \right)\left(2N_{T}{\left(z\right)}^{*}+T\beta^{T}\right)\frac{dz}{z}{\left(I_{n}-T\alpha \right)}^{-T}\\ \; & ={\left(I_{n}-T\alpha \right)}^{-1}\left(\frac{2}{\pi i}\oint_{|z|=1}N_{T}\left(z\right)N_{T}{\left(z\right)}^{*}\frac{dz}{z}+T^{2}\beta \beta^{T}\right){\left(I_{n}-T\alpha \right)}^{-T}\\ \; & ={\left(I_{n}-T\alpha \right)}^{-1}\left(4P_{T}+T^{2}\beta \beta^{T}\right){\left(I_{n}-T\alpha \right)}^{-T}\\ \; & =4{\left(I_{n}-T\alpha \right)}^{-1}P_{T}{\left(I_{n}-T\alpha \right)}^{-T}+\beta_{T}\beta_{T}^{T}. \end{array}$$

Here, (201) is used along with the relations

$$\oint_{|z|=1}N_{T}\left(z\right)\frac{dz}{z}=0,\;\oint_{|z|=1}N_{T}{\left(z\right)}^{*}\frac{dz}{z}=\oint_{|z|=1}N_{T}{\left(1/z\right)}^{T}\frac{dz}{z}=0$$

in view of the vanishing residues ${\mathrm{Res}}_{z=0}\frac{N_{T}\left(z\right)}{z}=0$ and ${\mathrm{Res}}_{z=\infty }\frac{N_{T}\left(1/z\right)}{z}=0$ by the structure of (200). Also, the last equality in (205) employs the relation

$$\beta_{T}=T{\left(I_{n}-T\alpha \right)}^{-1}\beta$$

between the input matrices of the worst-case noise shaping filter *G* in (159) and its discrete-time counterpart $G_{T}$ in (152). On the other hand, a combination of the second equality in (203) with (61) and (176) leads to

(206) $$J_{T}≼\underset{\omega \in R}{\sup}\Sigma \left(\omega \right)\frac{1}{2\pi }\int_{R}N\left(i\omega \right)N{\left(i\omega \right)}^{*}d\omega =2TP.$$

Therefore, by (205) and (206),

(207) $$4{\left(I_{n}-T\alpha \right)}^{-1}P_{T}{\left(I_{n}-T\alpha \right)}^{-T}+\beta_{T}\beta_{T}^{T}≼2TP.$$

The left and right multiplication of both sides of (207) by the matrices *L* and $L^{T}$, respectively, leads to

(208) $$L_{T}P_{T}L_{T}^{T}≼L_{T}P_{T}L_{T}^{T}+L\beta_{T}\beta_{T}^{T}L^{T}≼2TLPL^{T}.$$

Here, use is also made of the relation

$$L_{T}=2L{\left(I_{n}-T\alpha \right)}^{-1}$$

between the output matrices of the worst-case noise shaping filter *G* in (159) and its discrete-time counterpart $G_{T}$ in (152). The left and right multiplication of (208) by the symmetric matrix $M_{T}^{-1}$ yields

(209) $$\begin{array}{cc} \Xi_{T}\Xi_{T}^{T}\; & =\rho_{T}P_{T}\rho_{T}^{T}=M_{T}^{-1}L_{T}P_{T}L_{T}^{T}M_{T}^{-1}≼2TM_{T}^{-1}LPL^{T}M_{T}^{-1}\\ \; & =2TM_{T}^{-1}M\rho P\rho^{T}M^{T}M_{T}^{-1}=2TM_{T}^{-1}M\Xi \Xi^{T}M^{T}M_{T}^{-1}, \end{array}$$

where use is also made of the matrices $\Xi_{T}$, $\rho_{T}$ from (168) and (151) and Ξ, ρ from (181) and (162). By taking the trace on both sides of (209), it follows that

(210) $$\begin{array}{cc} ∥\Xi_{T}{∥}_{F}^{2}\; & ⩽2T\mathrm{Tr}(M_{T}^{-1}M\Xi \Xi^{T}M^{T}M_{T}^{-1})\\ \; & =2T\mathrm{Tr}(\Xi^{T}M^{T}M_{T}^{-2}M\Xi )\\ \; & ⩽2T\lambda_{\max}\left(M^{T}\Gamma^{-1}M\right){∥\Xi ∥}_{F}^{2}, \end{array}$$

with Γ from (157). Since the matrix $M^{T}\Gamma^{-1}M$ is isospectral to $\Gamma^{-1}MM^{T}=\Gamma^{-1}\Delta$ in view of (164), with the largest eigenvalue in (198), then (210) establishes the second inequality in (199), thus completing the proof. □

Since Theorem 6 involves the generalized spectrum of the matrices Δ and Γ which quantify the second-order moments of the innovation components of the worst-case disturbance in its continuous and discrete-time representations, we provide an upper bound for it as follows.

**Lemma** **1.** *The largest generalized eigenvalue (198) for the matrices* Γ*,* Δ *in (157) and (164), associated with the worst-case noise shaping filter in Theorem 4, satisfies*

(211) $$\sigma ⩽1+q\lambda_{\max}\left(\left(D^{T}D-D_{T}^{T}D_{T}\right)\Delta \right),$$

*where q is the parameter from (144).*

**Proof.**  From (157) and positive semi-definiteness of the solution $R_{T}$ of the ARE (140)–(142), it follows that

(212) $$\Gamma^{-1}=M_{T}^{-2}=I_{m}-qD_{T}^{T}D_{T}-B_{T}^{T}R_{T}B_{T}≼I_{m}-qD_{T}^{T}D_{T}.$$

Since Δ≻0 in (164), the matrix $\Gamma^{-1}\Delta$ is isospectral to $\sqrt{\Delta }\Gamma^{-1}\sqrt{\Delta }$ which, in view of (212), satisfies

(213) $$\begin{array}{cc} \sqrt{\Delta }\Gamma^{-1}\sqrt{\Delta }\; & ≼\sqrt{\Delta }\left(I_{m}-qD_{T}^{T}D_{T}\right)\sqrt{\Delta }\\ \; & =\sqrt{\Delta }\left(\Delta^{-1}+q\left(D^{T}D-D_{T}^{T}D_{T}\right)\right)\sqrt{\Delta }\\ \; & =I_{m}+q\sqrt{\Delta }\left(D^{T}D-D_{T}^{T}D_{T}\right)\sqrt{\Delta }. \end{array}$$

Due to monotonicity of the function $\lambda_{\max}\left(\cdot \right)$ on the subspace of real symmetric (or complex Hermitian) matrices with respect to the cone of positive semi-definite matrices, (213) implies that

$$\lambda_{\max}\left(\sqrt{\Delta }\Gamma^{-1}\sqrt{\Delta }\right)⩽1+q\lambda_{\max}\left(\sqrt{\Delta }\left(D^{T}D-D_{T}^{T}D_{T}\right)\sqrt{\Delta }\right),$$

which leads to (211) in view of the same similarity transformation and isospectrality argument as above. □

The inequality (197) remains valid if the geometric factor σ is replaced with its upper bound from (211). This inequality, or its equivalent form

$$\tau_{*}⩽\frac{2\sigma }{a_{\mathrm{temp}}}T,$$

provides an insight into the relation between the temporal part $a_{\mathrm{temp}}$ of the mean anisotropy level a>0 and the degree of distinguishability of the worst-case noise *W* from its martingale component MV in terms of the characteristic time $\tau_{*}$ for their discrepancy to manifest itself through relative entropy. This makes Theorem 6 applicable to a rational choice of the parameter *a* as the amount of statistical uncertainty in the external disturbance acting on the LCTI system.

## 11. Continuous-Time Anisotropic Norm Computation for RLC Circuit

**Figure 7:**
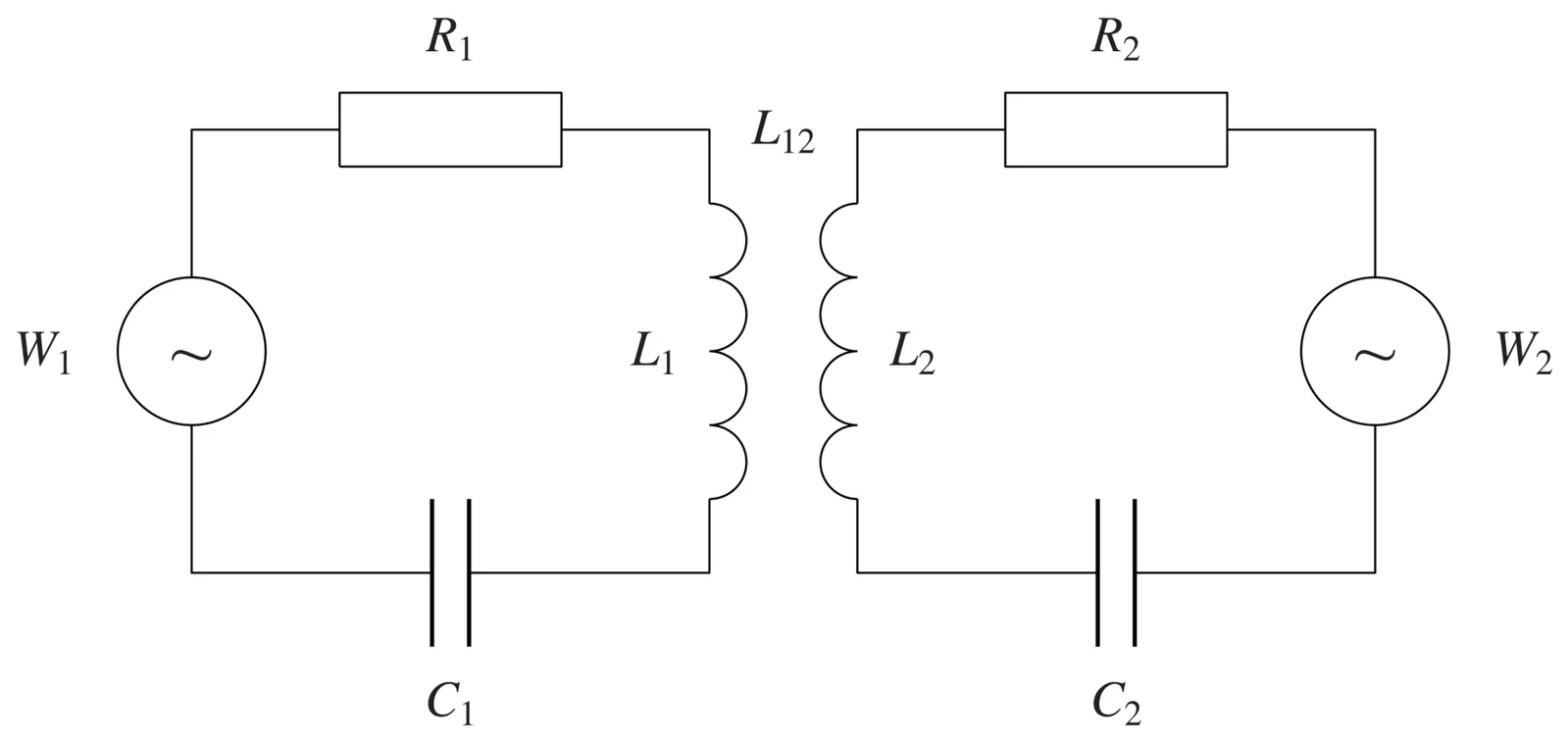
A two-loop RLC circuit with inductive coupling between the loops involving two noise voltage sources.

As a numerical illustration of the above results, consider a two-loop RLC circuit in Figure 7 with resistance, inductance and capacitance coefficients $R_{k},L_{k},C_{k}>0$ for the *k*th loop, k=1,2.

The circuit loops are inductively coupled, which is quantified by the mutual inductance coefficient $L_{12}$, so that the magnetic field energy stored by the coupled inductors is given by the quadratic form

(214) $$\frac{1}{2}\sum_{k=1}^{2}L_{k}J_{k}^{2}+L_{12}J_{1}J_{2}=\frac{1}{2}{∥J∥}_{\Pi }^{2},\;J:=\left[\begin{array}{c} J_{1}\\ J_{2} \end{array}\right].$$

Here, $J_{k}$ is the current in the *k*th loop, and use is made of an auxiliary positive definite matrix

(215) $$\Pi :=\left[\begin{array}{cc} L_{1} & L_{12}\\ L_{12} & L_{2} \end{array}\right].$$

The loops involve two noise sources (for example, of thermal or crosstalk origin [72,73]) with irregular voltages whose integrals over time (such as flux linkages) are represented by an $R^{2}$-valued Ito process

(216) $$W:=\left[\begin{array}{c} W_{1}\\ W_{2} \end{array}\right].$$

Accordingly, the integrals of the noise voltages are understood in the generalized sense since *W* is not differentiable with respect to time. The electric charges $Q_{k}$ at the capacitors have continuously differentiable sample paths with probability one, while their time derivatives

$${\dot{Q}}_{k}=J_{k}$$

are Ito processes. With $Q_{k}$ playing the role of generalized coordinates, the Lagrangian dynamics formalism along with (214) and Kirchhoff’s voltage law lead to a set of two SDEs

(217) $$L_{k}d{\dot{Q}}_{k}+L_{12}d{\dot{Q}}_{3-k}+\left(R_{k}{\dot{Q}}_{k}+\frac{1}{C_{k}}Q_{k}\right)dt=dW_{k},\;k=1,2,$$

which are coupled through the mutual inductance $L_{12}$. The SDEs (217) can be assembled into one SDE (11) for the state process *X*, given by

(218) $$X:=\left[\begin{array}{c} Q\\ \dot{Q} \end{array}\right],\;Q:=\left[\begin{array}{c} Q_{1}\\ Q_{2} \end{array}\right],$$

with the state and input dimensions n=4, m=2 in view of (216) and the state-space realization matrices

(219) $$A:=\left[\begin{array}{cc} 0 & I_{2}\\ -\Pi^{-1}E & -\Pi^{-1}R \end{array}\right],\;B:=\left[\begin{array}{c} 0\\ \Pi^{-1} \end{array}\right].$$

The latter are expressed in terms of the matrix Π from (215) and (also positive definite) matrices

(220) $$E:=\left[\begin{array}{cc} \frac{1}{C_{1}} & 0\\ 0 & \frac{1}{C_{2}} \end{array}\right],\;R:=\left[\begin{array}{cc} R_{1} & 0\\ 0 & R_{2} \end{array}\right].$$

The resulting system *F* is a stable linear dissipative stochastic Hamiltonian system [74,75], with Π, *E*, *R* playing the role of the generalized mass, stiffness and damping matrices, respectively, and the Hamiltonian

$$\frac{1}{2}{(∥Q∥}_{E}^{2}+∥\dot{Q}{∥}_{\Pi }^{2})=\frac{1}{2}{(∥Q∥}_{E}^{2}+∥\Pi \dot{Q}{∥}_{\Pi^{-1}}^{2})$$

describing the total electric and magnetic field energy stored by the capacitors and inductors of the RLC circuit. Here, the vector $\Pi \dot{Q}$, consisting of the flux linkages at the inductors, corresponds to the generalized momentum in accordance with the electromechanical analogy. The Hurwitz property of the matrix *A* in (219) is secured by the positive definiteness of the matrices Π and (220).

If the robust performance analysis of the RLC circuit is concerned, for example, with the sensitivity of the voltage $\frac{Q_{1}}{C_{1}}$ at the first capacitor and the voltage $R_{2}J_{2}=R_{2}{\dot{Q}}_{2}$ at the second resistor to the voltage noises, then the system output *Z* in (7) can be specified by the matrices

(221) $$C:=\left[\begin{array}{cccc} \frac{1}{C_{1}} & 0 & 0 & 0\\ 0 & 0 & 0 & R_{2} \end{array}\right],\;D:=0,$$

in accordance with (218). Indeed, an appropriately scaled version of the filtered output process $Z_{T}$ in (51) satisfies

$$\frac{1}{\sqrt{2T}}Z_{T}\left(t\right)=C\frac{1}{\sqrt{2T}}X_{T}\left(t\right)\overset{a.s.}{⟶}CX\left(t\right)=\left[\begin{array}{c} \frac{1}{C_{1}}Q_{1}\left(t\right)\\ R_{2}J_{2}\left(t\right) \end{array}\right],\;\mathrm{as}\;T\to 0+,$$

for any t>0 in view of (55), and thus asymptotically reproduces the first capacitor and second resistor voltages of interest.

With the following values of the circuit parameters (their physical dimensions are omitted for brevity)

$$\begin{array}{cc} R_{1} & =0.3190,\;R_{2}=0.6324,\\ L_{1} & =1.1235,\;L_{2}=2.2478,\;L_{12}=0.8593,\\ C_{1} & =0.4927,\;C_{2}=0.2501 \end{array}$$

in (215) and (219)–(221), the state-space realization of the system *F* being considered is given by

$$F↔\left[\begin{array}{cccccc} 0 & 0 & 1.0000 & 0 & 0 & 0\\ 0 & 0 & 0 & 1.0000 & 0 & 0\\ -2.5530 & 1.9227 & -0.4013 & 0.3041 & 1.2579 & -0.4809\\ 0.9760 & -2.5138 & 0.1534 & -0.3976 & -0.4809 & 0.6287\\ 2.0296 & 0 & 0 & 0 & 0 & 0\\ 0 & 0 & 0 & 0.6324 & 0 & 0 \end{array}\right].$$

The spectrum of the matrix *A* is

(222) S={−0.3077±1.9516i,−0.0917±1.0747i}

(so that the system is highly resonant with two lightly damped oscillatory modes), and the bounds in (89) are

$$\frac{1}{r(A)}=0.5062,\;r\left(A^{-1}\right)=0.9271.$$

The lower of these bounds (see the first relation in (89)) exceeds noticeably the time scale parameter

(223) T=0.0810

of the conformal correspondence which is used in what follows along with the cutoff frequency

Ω=12.3481

from (25). Therefore, the effective frequency range of the low-pas filter Φ in (22)–(24), which specifies the filtered input and output $W_{T}$, $Z_{T}$ for the RMS gain (63), is adequate for capturing the transient processes in the system *F*. The matrices (127)–(130) of its discrete-time counterpart $F_{T}$ take the form

$$F_{T}↔\left[\begin{array}{cccccc} 0.9685 & 0.0229 & 0.1545 & 0.0055 & 0.0078 & -0.0029\\ 0.0116 & 0.9690 & 0.0028 & 0.1545 & -0.0029 & 0.0039\\ -0.3890 & 0.2832 & 0.9074 & 0.0677 & 0.0958 & -0.0354\\ 0.1438 & -0.3832 & 0.0342 & 0.9084 & -0.0354 & 0.0479\\ 3.9953 & 0.0465 & 0.3135 & 0.0111 & 0.0158 & -0.0058\\ 0.0909 & -0.2423 & 0.0217 & 1.2069 & -0.0224 & 0.0303 \end{array}\right].$$

**Figure 8:**
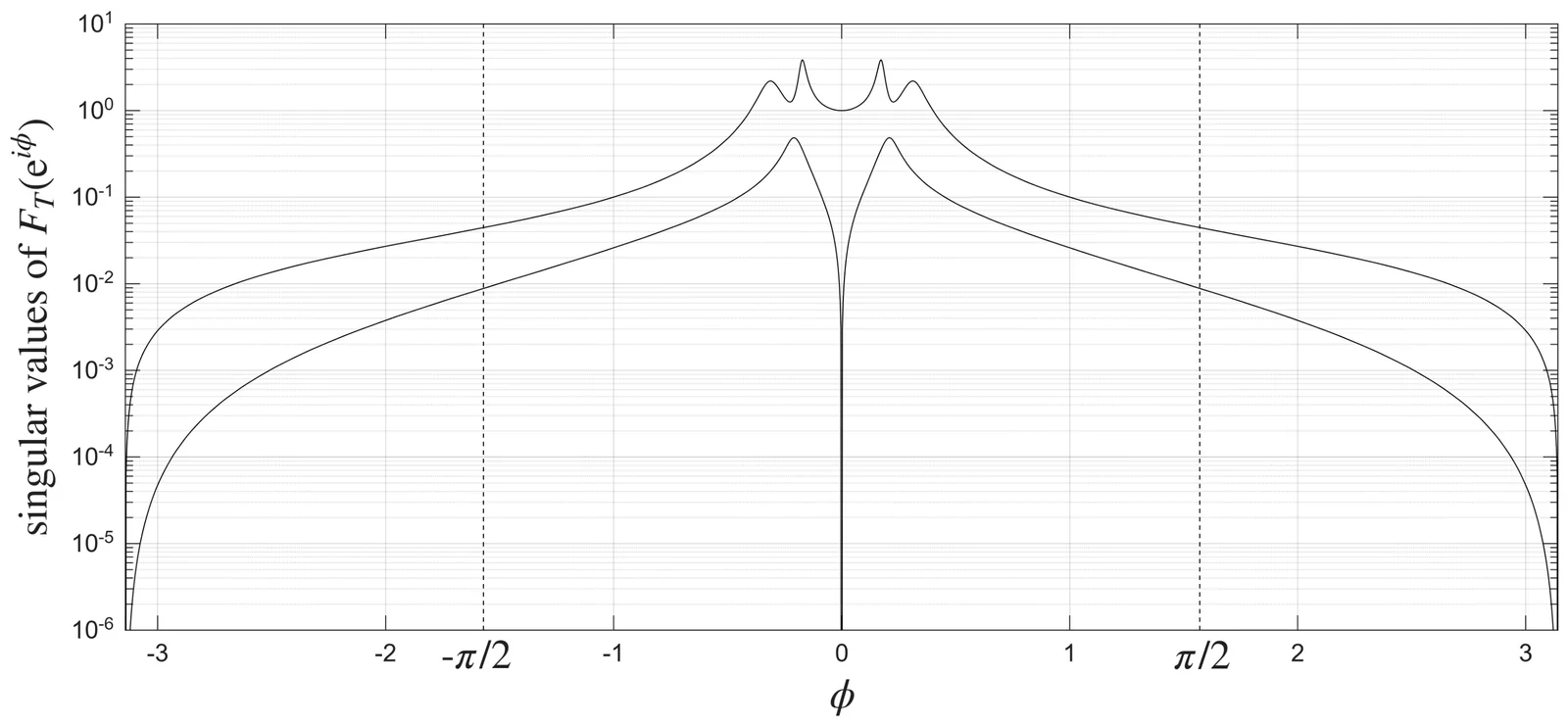
Singular values of the discrete-time transfer matrix $F_{T}\left(z\right)$ in (125) on the unit circle |z|=1 parameterized by $z=e^{i\varphi }$, with φ∈T from (85). The discrete-time frequency interval |φ|<π/2 corresponds to the continuous-time frequency interval (−Ω,Ω), where Ω is the cutoff frequency (25).

The behaviour of the singular values of the transfer function $F_{T}$ in (125) on the unit circle (see Figure 8) shows that the system $F_{T}$ is substantially nonround (and hence, so also is the system *F*).

Accordingly, there is a large gap between the scaled $H_{2}$-norm and the $H_{\infty }$-norm of the discrete-time system $F_{T}$:

$$\frac{1}{\sqrt{m}}∥F_{T}{∥}_{2}=0.4701,\;{∥F_{T}∥}_{\infty }=3.8617.$$

**Figure 9:**
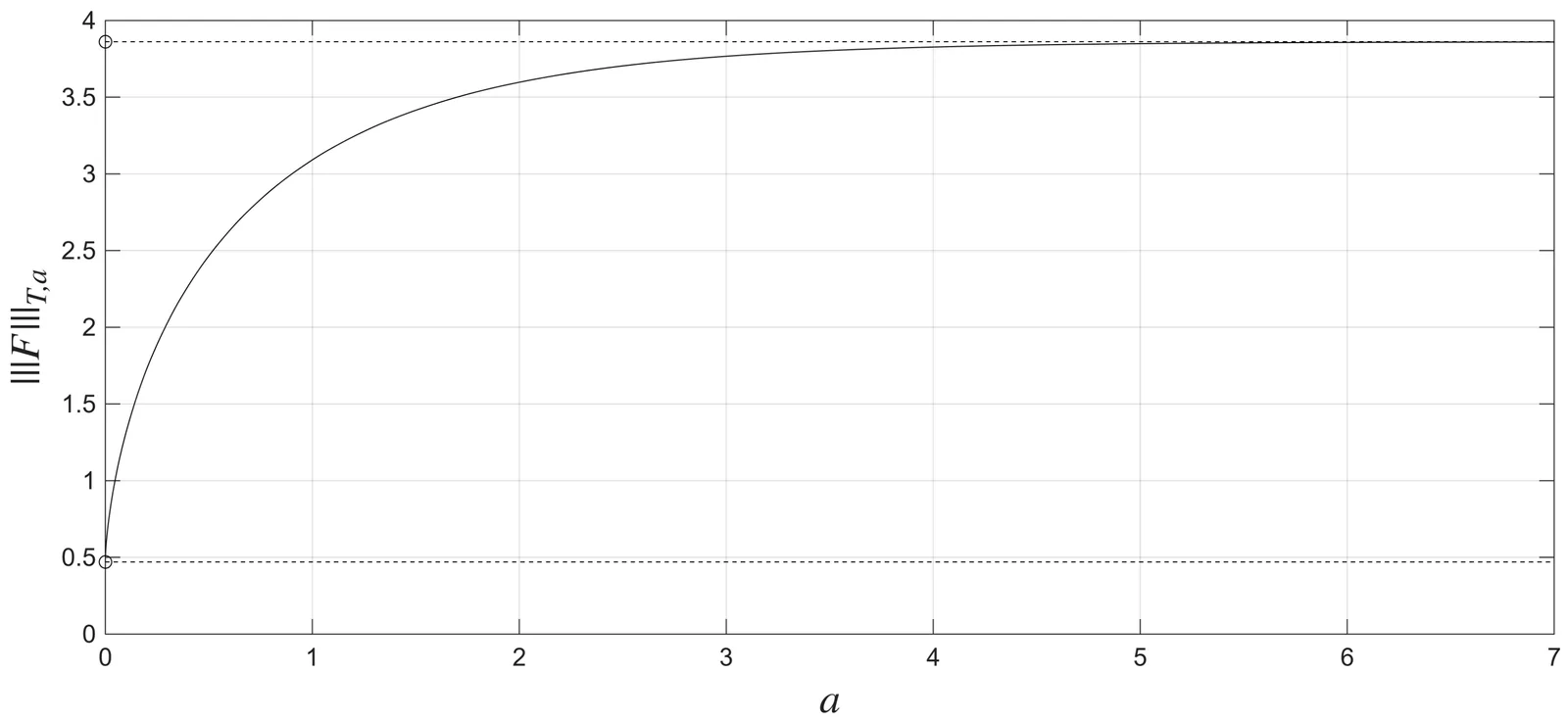
The two-parameter anisotropic norm $\left||\right|F{\left||\right|}_{T,a}$ (represented by a solid line) of the LCTI system *F*, associated with the RLC circuit, as a function of the input mean anisotropy level a⩾0 for the discrete-time counterpart $F_{T}$ of the system. The dashed lines represent its extreme values $\frac{1}{\sqrt{m}}{∥F_{T}∥}_{2}$ and $∥F_{T}{∥}_{\infty }$.

These norms are the endpoints of the range of the (T,a)-anisotropic norm $\left||\right|F{\left||\right|}_{T,a}$ of the system (as the mean anisotropy level *a* varies from 0 to +∞), computed using Theorem 4 (with Newton’s iterations [Section 4] in [18], [Section 9] in [21]) and shown in Figure 9.

For example, at

(224) a=0.5000,

the norm being considered is

$$\left||\right|F{\left||\right|}_{T,a}=2.4650,$$

and the matrices (147) of the worst-case noise shaping filter (158) are given by

(225) $$L=\left[\begin{array}{cccc} 0.0697 & 0.0861 & 0.2543 & 0.2487\\ -0.0422 & 0.0009 & 0.1163 & 0.2334 \end{array}\right],\;M=\left[\begin{array}{cc} 1.0000 & -0.0002\\ 0.0002 & 1.0000 \end{array}\right].$$

In comparison with (222), the spectrum of the corresponding matrix α in (160) is closer to the imaginary axis:

{−0.2528±1.9485i,−0.0011±1.0659i},

so that the parasitic feedback loop in Figure 2 leads to a less stable system (20). While the matrix *L* is relatively small, *M* is close to the identity matrix $I_{2}$. Moreover, since D=0 in this example as indicated in (221), the matrix *M* is orthogonal in view of (164), although its orthogonality is slightly distorted in (225) by the finite accuracy of digital representation. The resulting martingale part MV of the worst-case noise *W* has the identity diffusion matrix

$$\Delta =I_{2}$$

and thus inherits from *V* the property of being a standard Wiener process in $R^{2}$. In the worst-case noise scenario at the mean anisotropy level (224), the spatial and temporal components in (165)–(167) are

(226) $$a_{\mathrm{spat}}=0.0752,\;a_{\mathrm{temp}}=0.4248.$$

The spatial component comes from the covariance matrix (155) of the auxiliary stationary Gaussian sequence (100), which is also the invariant covariance matrix of the filtered version $W_{T}$ of *W*:

(227) $$L_{T}P_{T}L_{T}^{T}+M_{T}^{2}=\lim_{t\to +\infty }\mathrm{cov}\left(W_{T}\left(t\right)\right)=\left[\begin{array}{cc} 1.9948 & 0.5508\\ 0.5508 & 1.3801 \end{array}\right]$$

(its eigenvalues are 2.3182 and 1.0567). The KL relative entropy rate (180) of the worst-case noise *W* with respect to its martingale part MV, and the characteristic time scale (194) for their discrepancy to manifest itself are

(228) $$\frac{1}{2}{∥\Xi ∥}_{F}^{2}=4.1316,\;\tau_{*}=0.2420.$$

The geometric factor in (198) is

σ=0.9873,

and its upper bound in (211) is 1.0000. In addition to (226), the intermediate term in (199) and the right-hand side of (197) are

$$\frac{1}{2}{∥\Xi_{T}∥}_{F}^{2}=0.6431,\;2\sigma \frac{T}{\tau_{*}}=0.6607.$$

**Figure 10:**
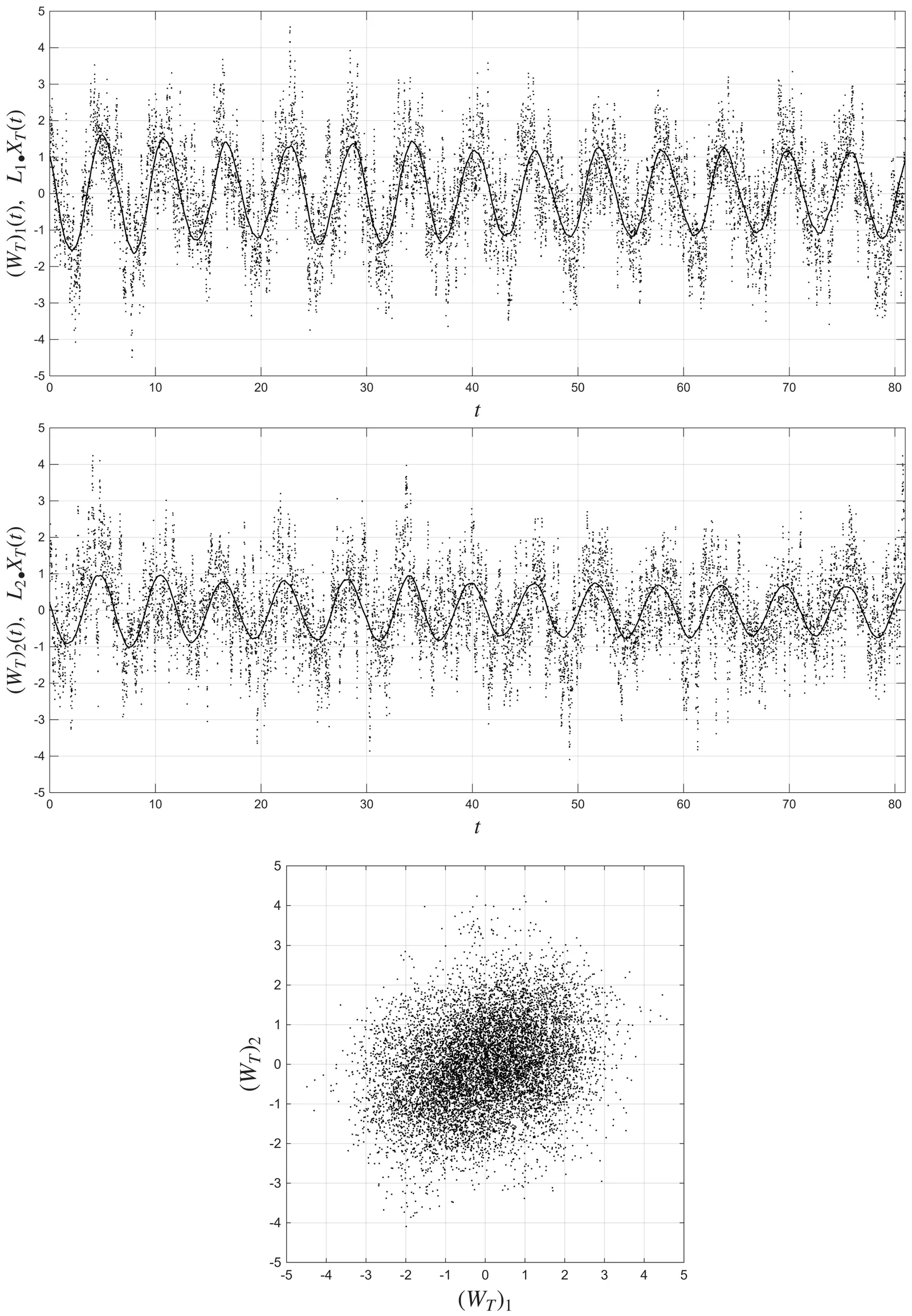
The entries of the filtered version $W_{T}$ (the dotted lines with time step 0.0081) of the worst-case noise *W* for the RLC circuit, shown along with the drift $LX_{T}$ (represented by the solid lines).

**Figure 11:**
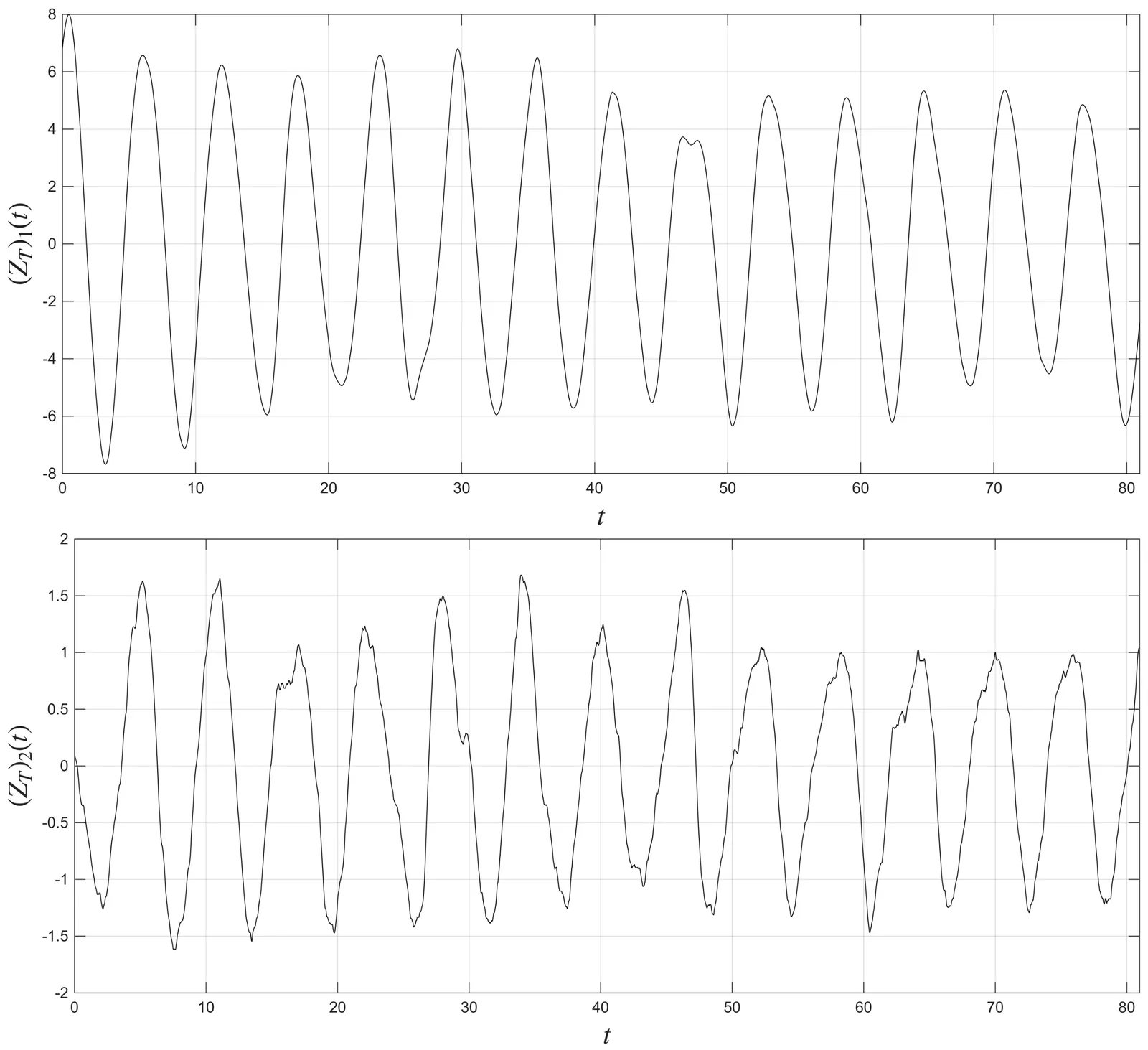
The entries of the filtered version $Z_{T}$ of the output *Z* of the RLC circuit subject to the worst-case noise *W*.

The time scale $\tau_{*}$ in (228) noticeably (yet insignificantly) exceeds the Tustin transform parameter *T* in (223), with $\frac{\tau_{*}}{T}=2.9887$. In this sense, the worst-case noise at the mean anisotropy level (224) can informally be qualified as moderately coloured, while comparison of $\tau_{*}$ with other time scales can also be considered. Typical sample paths of the filtered versions

$$W_{T}=LX_{T}+MV_{T},\;Z_{T}=\left(C+DL\right)X_{T}+DMV_{T}$$

of the noise process *W* and the output signal *Z* of the RLC circuit are provided in Figure 10 and Figure 11 along with those of $LX_{T}$.

They are obtained by the numerical integration of the SDE

$$d\left[\begin{array}{c} X_{T}\\ V_{T} \end{array}\right]=\left[\begin{array}{cc} \alpha & \beta \\ 0 & -\Omega I_{m} \end{array}\right]\left[\begin{array}{c} X_{T}\\ V_{T} \end{array}\right]dt+\left[\begin{array}{c} 0\\ \sqrt{2\Omega }I_{m} \end{array}\right]dV$$

(which combines the ODE (202) with the SDE (22) and uses the matrices (160)) in such a way that the probability law of the jointly Gaussian processes $X_{T}$, $V_{T}$ in their stationary regime is exactly reproduced. Note that the elliptical shape and tilt of the scatter plot in Figure 10 is in accordance with the eigenstructure of the invariant covariance matrix (227), including the positive correlation between the components of $W_{T}$ considered at the same moment of time.

## 12. Concluding Remarks

We have outlined a particular way of extending the discrete-time anisotropy-based criteria to LCTI systems governed by SDEs with statistically uncertain Ito processes as external noise inputs. This approach uses the RMS gain of the system in terms of filtered versions of the input and output processes. The time scale *T* of the low-pass filter parameterizes Tustin’s transform of the original LCTI system *F* to its LDTI counterpart $F_{T}$ with the same RMS gain, though understood in the usual sense for fragments (or steady-state RMS values) of stationary Gaussian random sequences.

The resulting two-parameter norm $\left||\right|F{\left||\right|}_{T,a}$ is defined as the *a*-anisotropic norm $\left||\right|F_{T}{\left||\right|}_{a}$ of the effective discrete-time system, with the input mean anisotropy threshold *a* quantifying indirectly the deviation of the spectral density of the Ito process at the input of the original system from constant scalar matrices. The computation of this norm reduces to the numerical solution of a set of matrix algebraic Riccati, Lyapunov and log-determinant equations developed 30 years ago in the discrete-time anisotropy-based control theoretic framework. Under Tustin’s inverse transform, the worst-case disturbance inherits the general feedback structure, whereby its drift depends linearly on the internal state of the system.

Therefore, in combination with Tustin’s direct and inverse transforms, the methods of anisotropy-based robust performance analysis and control design, developed earlier for discrete-time systems, are applicable to continuous-time settings. In particular, this allows the state-space solutions [19,23] of discrete-time anisotropy-based control and filtering problems to be converted to their continuous-time counterparts. However, such extension is limited to infinite-horizon settings for time invariant systems since it substantially employs the commutativity between two LCTI systems as input-output operators, provided one of them has a scalar transfer function. Accordingly, an extension of finite-horizon time-varying formulations of the discrete-time anisotropy-based theory to their continuous-time counterparts requires the development of alternative approaches, which will be reported elsewhere.

## Figures and Tables

**Figure 3:**
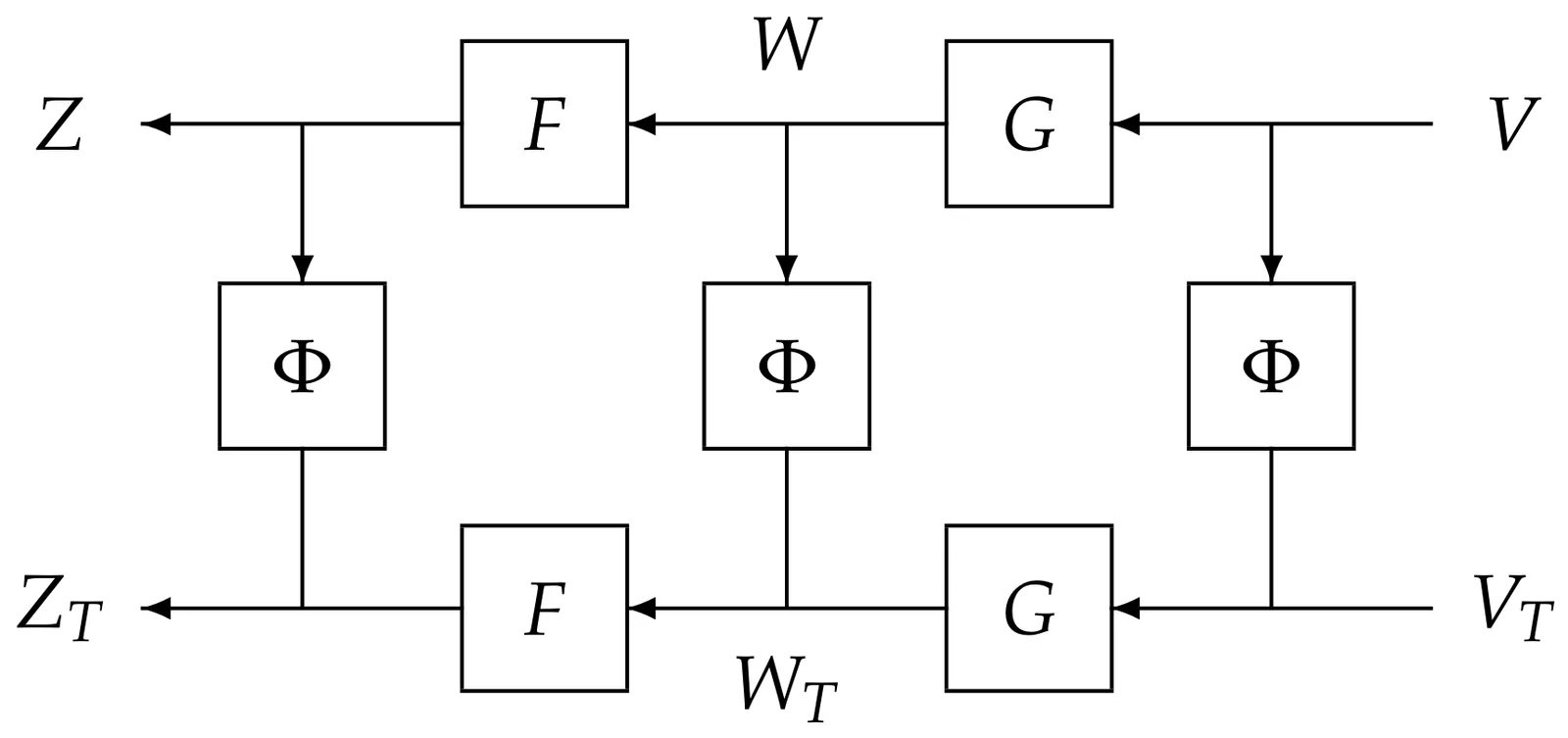
The LCTI system *F* with the output *Z* and input *W* produced by an LCTI noise shaping filter *G* from the standard Wiener process *V*, and their filtered versions $Z_{T}$, $W_{T}$, $V_{T}$ obtained using the low-pass filter Φ. The processes $Z_{T}$, $W_{T}$, $V_{T}$ are related by the same input-output operators *F*, *G* as *Z*, *W*, *V* (cf. (42)–(50)).

## Data Availability

Data are contained within the article.

## Abbreviations

The following abbreviations are used in this paper:

ALE	algebraic Lyapunov equation
ARE	algebraic Riccati equation
KL	Kullback-Leibler
LCTI	linear continuous time invariant
LDTI	linear discrete time invariant
ODE	ordinary differential equation
OU	Ornstein-Uhlenbeck
PDF	probability density function
RLC	resistance, inductance, capacitance
RMS	root-mean-square
SDE	stochastic differential equation
